# Association of Recent and Long-Term Supplement Intakes With Laboratory Indices in Patients With COVID-19 in Tehran, Iran, During 2020

**DOI:** 10.3389/fnut.2022.834826

**Published:** 2022-06-06

**Authors:** Rezvan Hashemi, Mohsen Montazer, Zahra Salehi, Leila Azadbakht

**Affiliations:** ^1^Department of Geriatric Medicine, Ziaeian Hospital, Tehran University of Medical Sciences, Tehran, Iran; ^2^Department of Community Nutrition, School of Nutritional Sciences and Dietetics, Tehran University of Medical Sciences, Tehran, Iran; ^3^Diabetes Research Center, Endocrinology and Metabolism Clinical Sciences Institute, Tehran University of Medical Sciences, Tehran, Iran; ^4^Department of Community Nutrition, School of Nutrition and Food Science, Isfahan University of Medical Sciences, Isfahan, Iran

**Keywords:** supplement, COVID-19, BUN, ICU, general ward, creatinine, CRP

## Abstract

**Background:**

Although previous studies observed the relationship between individual dietary supplements and enhancing body resistance against viruses, few studies have been conducted regarding the role of different supplements in treatment of COVID-19. This article aims to determine the association of recent and long-term supplement consumption on the biochemical indices and impatient duration among patients with COVID-19.

**Methods:**

In this cross-sectional study on 300 adult men and women with COVID-19, recent and long-term supplement intakes were investigated by using a questionnaire. In addition, lifestyle was also assessed in aspects of fruits and vegetable consumption, physical activity, sleeping duration, fluid intake, and smoking status. Furthermore, the laboratory and paraclinical parameters were obtained from medical records. The relationship between supplement intake with the length of hospitalization and clinical laboratory tests was investigated by one-way analysis of variance (ANOVA).

**Results:**

Those patients with supplement intake in the last 2 months had a significantly lower amount of blood urea nitrogen (BUN) (31.31 ± 13.87 vs. 37.57 ± 19.77 mg/dL, *P*: 0.002) and higher serum 25(OH)D (28.13 ± 14.09 vs. 23.81 ± 13.55 ng/mL, *P*: 0.03). Subjects with long-term supplement intake had a significantly lower invasive oxygen support (0.00 vs 5.10 %, *P*: 0.05), lactate dehydrogenase (LDH) (498.11 ± 221.43 vs. 576.21 ± 239.84 U/L, P: 0.02), fewer days of fever (0.49 ± 3.54 vs. 2.64 ± 9.21, *P*: 0.02), and higher serum 25(OH)D (31.03 ± 13.20 vs. 22.29± 13.42 ng/mL, *P* < 0.001). The length of hospital stay was practically the same between groups who received and did not receive supplementation during the 2 months prior to hospitalization (6.36 ± 3.32 vs. 6.71 ± 4.33 days, *P*: 0.004). Similarly, people who took supplements during the past year had practically similar hospitalization lengths (6.29 ± 4.13 vs. 6.74 ± 3.55 days, *P*: 0.004).

**Conclusion:**

In conclusion, although practically the length of hospital stay was the same in both groups of supplement consumers and others, immune-boosting supplements were associated with improved several laboratory indices. However, due to the cross-sectional nature of our study, further longitudinal studies seem to be essential.

## Introduction

The new variation of coronavirus, SARS-CoV-2 (severe acute respiratory syndrome coronavirus 2), which triggers COVID-19, is undoubtedly the foremost dangerous coronavirus with the greatest morbidity and death rate. Human society experienced the most important acute health trouble due to the coronavirus pandemic in recent year (1). The crude mortality ratio is 1.63% based on Worldometer by January 21, 2022 (2), which can vary in different countries depending on age and the presence of comorbidities (1).

As of January 21, 2022, 9,807,128,664 doses of the COVID-19 vaccine have been administered worldwide, according to covidvax; however, maintaining social distancing and carrying face masks are recommended in public spaces to avoid the transmission of COVID-19 (3). Furthermore, lifestyle factors, including dietary intake, exercise, smoking, alcohol intake, screen time, and sleep, may also additionally have a crucial influence on COVID-19 vulnerability (4). Hence, many researchers highlighted the importance of taking suitable dietary interventions and nutritional supplements to boost our immune systems. The relationship between taking protein, omega-3, vitamin A, B complex, C, D, E, minerals like zinc, selenium, iron, copper, and magnesium supplements and improving the immune system has been investigated (5–16). Previous studies have also investigated the effects of glycophosphopeptical AM3 on respiratory diseases (17, 18). Furthermore, a review study has discussed the potential prophylactic and therapeutic benefits of glycophosphopeptical AM3 on COVID-19 (19). Vitamin D, lactoferrin, and selenium supplementation have possibly desirable effects on COVID-19 prevention (11, 20–25). It is recently reported that D3 intake for a few weeks at 10 000 IU/d followed by 5,000 IU/d to reach the minimum of 150 nmol/L for 25(OH)D serum is a prophylactic measure to COVID-19 for high-risk people (8). The researchers recently reported vitamin D, zinc, omega-3, vitamin C, selenium, magnesium, lactoferrin, L glutamine, gut microbiota, nigella sativa, and propolis supplementation could be considered as possible COVID-19 treatments (15, 22, 25–33). Early dietary supplementation in suspicious COVID-19 cases can contribute to better treatment results, and good nutritional status is essential prior to the other medical measures (33).

The necessity of immune-boosting supplementation is one of the most common ambiguities during the COVID-19 pandemic. Furthermore, in patients with COVID-19, possible differences in length of hospital stay and biochemical parameters in people who took the supplements with others are yet to be determined. In general, the benefits of taking immune-boosting supplements during the COVID-19 pandemic are not completely conclusive. Previous studies have observed relationships between appropriate nutritional status and enhancing body resistance against viruses (33–35). However, the information about supplementation as a preventive and treatment measure for COVID-19 remains controversial. Although there are reports on patients with COVID-19 nutritional supplement effects, there is no study on multi-dietary supplement's role in the prevention and treatment of COVID-19 simultaneously. This article aims to determine the association of recent and long-term supplement consumption before disease incidence on the biochemical indices and impatient duration among infected patients to COVID-19.

## Materials and Methods

### Study Design and Population

The main aim of this cross-sectional study was to investigate the association of recent and long-term supplement intakes with laboratory indices and length of hospital stay in hospitalized patients with COVID-19. Participants were recruited from patients with COVID-19 hospitalized in Ziaeian hospital affiliated with Tehran University of Medical Sciences. The data collection process was performed from March 24, 2020, to November 20, 2020. This study was performed on 300 adults aged 18 years and older admitted to Ziaeian Hospital who were randomly selected. Subjects were confirmed patients with COVID-19 diagnosed by the general practitioner (GP) regarding laboratory results, lungs CT scan, and clinical symptoms simultaneously. Consumption of dietary supplements in the last 2 months and the past year was classified into recent and long-term supplement intakes, respectively. Supplement intake modulating the immune system function, such as A, C, D, E, and B vitamins, zinc, selenium, iron, omega-3, and protein, as well as probiotics, was studied. Written informed consent was obtained from all participants. Inclusion criteria were as follows: no pregnancy, no lactation, and no serious condition, such as dementia, history of a large stroke, and active cancer within 5 years. Patients with non-communicable diseases, such as diabetes, hypertension, hyperlipidemia, hypothyroid, kidney disease, and fatty liver, were included. This study was approved by the ethical committee of Tehran University of Medical Sciences with the ethics code IR.TUMS.VCR.REC.1399.036 and grant number 99-1-212-47266.

### Assessment of Anthropometric and Obesity Characteristics

Anthropometric parameters, such as weight, height, and body mass index of patients, were measured at admission. Weight was measured with light clothing and using a digital scale with an accuracy of 100 grams, and height was also obtained using a stadiometer with an accuracy of 0.1 cm after removing the shoes. To reduce possible errors, all these measurements were performed by a healthcare staff. Using a demographic questionnaire, patient sudden and severe weight changes in the last month were assessed. Moreover, to evaluate weight changes during hospitalization, subjects' weight was measured at the time of discharge. Finally, body mass index was computed by dividing weight (kg) to the square of height (m^2^).

### Assessment of Usual Dietary and Supplement Intake

Daily energy intake, and macro- and micronutrients of the subjects were obtained using 24-h recall based on the number of hospitalization days during hospital stay. Using USDA modified food composition table for Iranian foods, the daily macro- and micronutrient intake of each patient was calculated (36). Patients were evaluated regarding following a special diet before the onset of the disease, servings of fruit consumption per day during the past year, number of daily main meals received, type and amount of bread, meat, dairy, and oil consumed, and the frequency of fast-food consumption. Moreover, the amount of main meals and snacks consumed during hospitalization in comparison with the amount consumed before the onset of the disease was obtained using questionnaire. In addition, fluid intake habits regarding the frequency of carbonated beverages consumption and daily water intake were assessed based on questionnaire data. Finally, to assess the nutrition status of each participant, an overall nutrition score was applied. We obtained the overall nutrition score for each participant by summing the scores of the type of meat consumed, the type of bread consumed, the amount of dairy consumed, the amount consumed of fruits and vegetables, and the consumption of sweetened carbonated beverages. The points allocated were as follows: consumption of red meat 0, chicken 1, fish 2, consumption of <1 glass of dairy 0, 1 glass of dairy 1, 2 glass of dairy 2, 3 glass of dairy 3, >3 glass of dairy 4, consumption of carbonated beverages 0 and non-consumption 1, consumption of white bread 0 and whole wheat bread 1, consumption of 1 unit of fruits 1, 2 units of fruits 2, 3 units of fruits 3 and consumption of <1 unit of vegetables 0, 1 unit of vegetables 1, 2 units of vegetables 2, 3 units of vegetables 3, and >3 units of vegetables 4 points were assigned. To evaluate the recent intake of supplements, we considered taking supplements during 2 months before hospitalization. We also obtained supplement intake during 1 year before admission to assess long-term supplementation. Long-term (over 1 year) and more recently (over the last 2 months) vitamin, mineral, probiotic, omega-3s, and protein supplement intake have been evaluated, and subjects have been asked to list their possible consumed supplements and state the received dose. Using supplement intake questionnaire, received supplements involved in the immune system function, such as A, C, D, E, and B vitamins, zinc, selenium, iron, omega-3, and protein, as well as probiotics, were obtained.

### Assessment of Comorbidities and Medications

Patient current comorbidities, such as diabetes, hypothyroid, asthma, arthroses, Alzheimer's disease, rheumatoid arthritis, chronic obstructive pulmonary disease, end-stage renal disease, Parkinson's, kidney failure, hepatitis B, meningitis, human immunodeficiency virus, cerebral vascular accident, chronic kidney disease, mental disease, obesity, and immune system deficiency, were obtained using medical records. In addition, based on medical record data, cardiovascular disease risk factors, including hypertension, hyperlipidemia, ischemic heart disease (IHD), deep vein thrombosis (DVT), congenital heart defects (CHD), coronary artery disease (CAD), and congestive heart failure (CHF), were assessed. Likewise, using questionnaire, a history of heart valve replacement, dialysis, coronary artery bypass graft surgery (CABG), liver surgery, chemotherapy, and various types of transplants were obtained. Furthermore, received medications during the hospitalization period with emphasis on immune system suppressors, such as corticosteroids and their dosage, were extracted from the questionnaire. Patients were also evaluated in terms of antiviral drugs azithromycin, heparin, oseltamivir, Kaletra, ribavirin, favipiravir, remdesivir, atazanavir, and ivermectin consumption based on medical record data. In addition to current diseases and drugs, a history of diseases and drugs was also obtained regarding medical records. The history of lipid-lowering, anticoagulant, psychiatry, statin, angiotensin-converting enzyme inhibitors (ACEIs), and angiotensin II receptor blocker (ARB) drugs were assessed using the questionnaire. The possible underlying disease, as well as the severity of the disease, was stated in the questionnaire based on the paraclinical findings in the medical records.

### Assessment of Clinical and Paraclinical Findings

Laboratory clinical tests were performed at the time of hospital admission using conventional methods, such as routine blood tests, cardiometabolic factors, renal function, liver enzymes, coagulation factors, and inflammation profile. The serum levels of important laboratory and paraclinical parameters, including blood urea nitrogen (BUN), creatinine, albumin, ferritin, 25(OH)vitamin D, c-reactive protein (CRP), d-dimer, and hemoglobin, were extracted using a questionnaire. In addition, according to medical records, other paraclinical findings in terms of aspartate aminotransferase (AST), alanine aminotransferase (ALT), and alkaline phosphatase (ALKPH) hepatic enzymes, electrolytes, creatine phosphokinase (CPK), lactate dehydrogenase (LDH), fasting blood sugar (FBS), HCO3, pH, partial pressure of oxygen (PO2), partial pressure of carbon dioxide (PCO2), and troponin serum levels were obtained. To measure CRP, troponin, ferritin, sodium, potassium, albumin, AST, ALT, LDH, creatinine, and urea, Abbott's Alinity Systems (Abbott, Chicago, IL, USA) were applied on plasma samples added with lithium heparin (Becton Dickinson, Franklin Lakes, NJ, USA). FBS levels were determined using the glucose oxidase-phenol 4-aminoantipyrine peroxidase (GOD-PAP) colorimetric method. Routine blood factors, such as white blood cell (WBC), red blood cell (RBC) and platelet count, neutrophils, lymphocytes and HCT percentage, mean corpuscular volume (MCV), mean corpuscular hemoglobin (MCH), and erythrocyte sedimentation rate (ESR), were obtained from anticoagulated blood samples with ethylenediaminetetraacetic acid tripotassium (K3 EDTA; Becton Dickinson, Franklin Lakes, NJ, USA) using Sysmex XN hematology analyzer and reagents (Sysmex Corporation, Kobe, Japan). Partial thromboplastin time (PTT), partial prothrombin time (PT), international normalized ratio (INR), and D-dimer were measured by benchtop analyzer STA compact Max 3 (Stago, Asnières-sur-Seine, France) and reagents on serum evacuated to 3.2% sodium citrate coagulation tubes (Becton Dickinson, Franklin Lakes, NJ, USA). The patient's symptoms, such as general pain, headache, cough, sore throat, joint pain, respiratory problems, anorexia, runny nose, lethargy, diarrhea, and vomiting, were documented in a questionnaire based on the evidence in the patient medical records.

### Assessment of Other Variables

Related data on sociodemographic variables, including age, sex, education, occupation, residency, family status, and marital status, were extracted from the demographic questionnaire. Female participants were assessed in aspects of the physiologic condition, such as pregnancy, lactation status, number of deliveries, and menopausal status, additionally. Lifestyle-related variables, including smoking status, current and past history of alcohol consumption and drug abuse, daily physical activity, facing stressful events in the last 6 months, and sleep duration changes, were obtained using a questionnaire. Finally, using questionnaire, the method of diagnosis of COVID-19 disease, the extent of contact with COVID-19 patients before infection, inpatient or non-hospital treatment, place of hospitalization, duration of hospitalization in addition to general condition at the time of discharge from the hospital were assessed.

### Statistical Analysis

**Figure 1:**
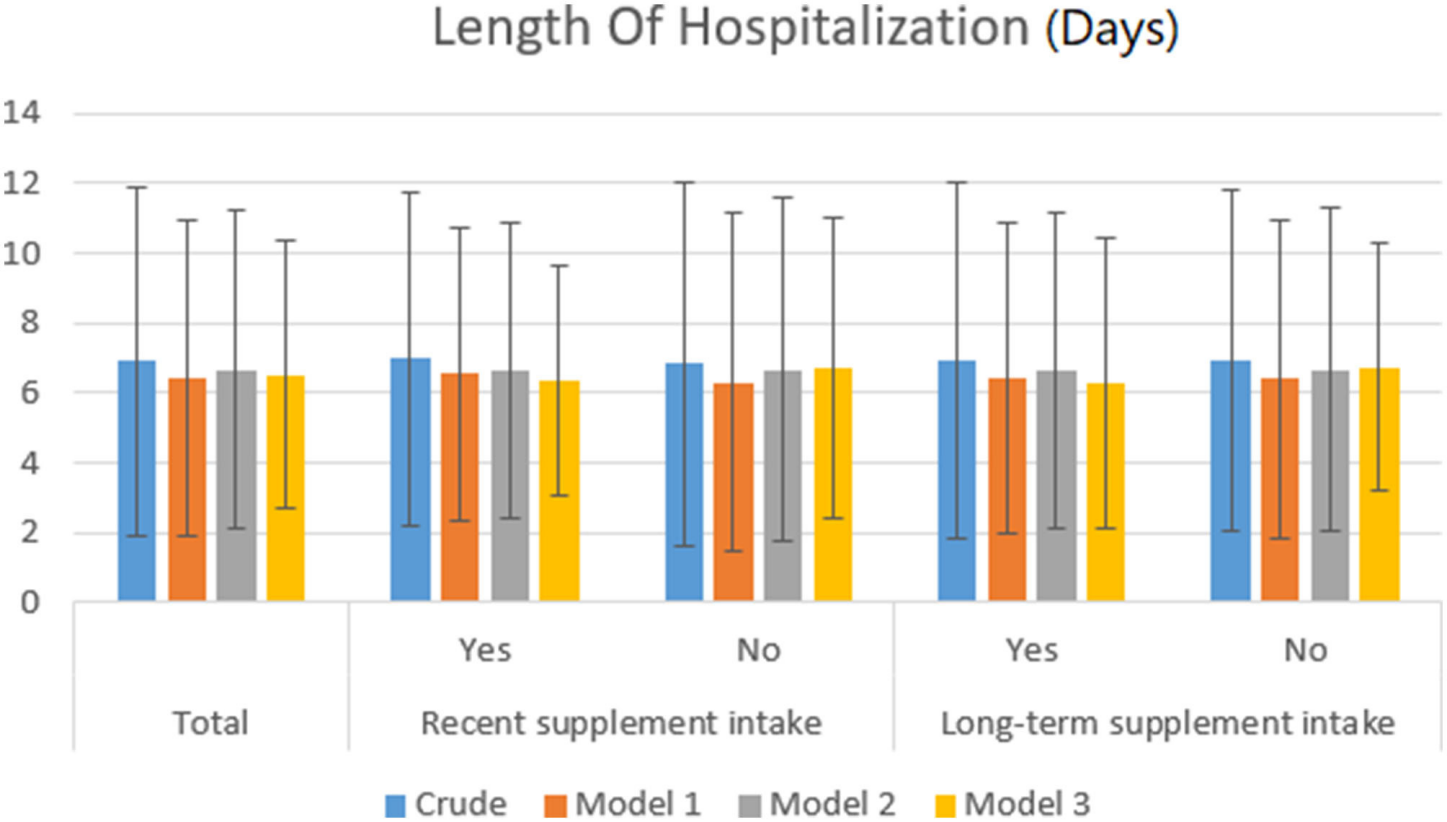
Length of hospitalization in different supplement intake categories.

Participants were categorized regarding recent and long-term supplement consumption. We applied one-way ANOVA and chi-square tests for continuous and categorical variables, respectively, to compare sociodemographic characteristics across different supplement intake categories. The relationship between more recent and long-term supplement intakes with the length of hospitalization was assessed using one-way ANOVA. Similarly, the association of recent and long-term supplementation with clinical laboratory tests was investigated by one-way ANOVA. By applying the chi-square test, recent and long-term supplement consumption relationships with the symptoms of disease (fever, cough, headache, runny nose, and sore throat) were examined. Moreover, the prevalence of various comorbidities and medications across different categories of supplement intake was assessed using the chi-square test. Potential confounders which were likely to affect the outcomes of our study were adjusted in three phases using ANCOVA. One of the factors that may affect the laboratory indices and duration of hospitalization is medication. We examined the interaction of drugs with supplements in such a way that in a separate model, we performed statistical adjustments. If the effect of the interaction of drugs with supplements was significant, then the effect would disappear. In model 1, variables of gender, age, history and the current onset of diseases, and history and current medications were adjusted. The length of hospitalization in different categories of supplement intake following gender, age, history and current onset of diseases, and history and current medications adjustment is presented in model 1 (Figure 1). Furthermore, in model 2, the *P*-values were controlled by body mass index, hemoglobin, and nutrition overall score, additionally, which were included as covariates (Figure 1). Finally, in model 3, multiple variable adjustments were performed and the existence of respiratory support, fever, blood oxygen level, partial pressure of carbon dioxide, albumin, 25(OH)D, and c-reactive protein serum levels were included as covariates, additionally.

## Results

Out of 300 subjects in the present study, 164 and 138 patients had recent and long-term supplement intake, respectively. 54.7% of the participants were males and 45.3% were females with a mean age of 51.95 ± 15.34 years. Table 1 and Supplementary Table 1 represent the sociodemographic characteristics of patients with COVID-19 who were included in the present study. People who have been supplemented in the last 2 months were younger, slightly higher educated, obese, and more stressed than those who did not take the supplement recently. Similarly, people who received supplements in the past year were people with younger age, more percentage of women, slightly more traveling experience, more sleep-alteration, and more stressful events than those who did not receive the supplement. There was no significant difference in marital status, the number of family members, job classification and comorbidities except for obesity in the two groups of recent supplementation and non-recent supplementation. No significant difference was observed in marital status, number of family members, job classification and comorbidities except for immune deficiency and asthma across different long-term supplementation groups. Anthropometric and obesity characteristics of patients with COVID-19 across recent and long-term supplementation categories are presented in Table 2. In people who have recently taken supplements, more weight changes have been observed, approaching the marginal significance (*P* = 0.07).Similarly those subjects with recent supplement intake showed significantly more prevalence of obesity (*P* = 0.04). Supplementation in recent years has been associated with less weight change (*P* = 0.002). History of different diseases and diagnosis methods among patients with COVID-19 are presented in Supplementary Table 2. Dietary intake during hospitalization among patients with COVID-19 in different supplement categories intake has been provided in Supplementary Table 3. There was a significant difference in the amount of breakfast and dinner consumption of those who took the supplement recently and those who did not take the supplement recently (*P* =0.004, 0.04). The use of different drugs did not differ much between the two groups. No significant difference was found in terms of dietary intake and medication in those who took or did not take supplements in the past year. Supplementary Table 4 illustrates the usual dietary intake among patients with COVID-19 in different supplement categories intake. There was a significant difference in the frequency of fast food consumption, consumption of lipid-lowering drugs and statins in those subjects with recent supplements compared to those who did not take supplements recently. Beverage and anticoagulants consumption was significantly different in those who did and did not receive supplementation in the past year (*P* = 0.05, 0.01). Biochemical indices in different supplement intake categories are provided in Supplementary Table 5. Alkaline phosphatase (146.64 ± 67.21 vs. 176.96 ± 143.10 IU/L, *P*: 0.03) and MCV (84.70 ± 6.56 vs, 86.39 ± 5.19 fl, *P*: 0.02) were significantly lower in the group that had taken the supplement in the last 2 months. Subjects with long-term supplement intake had significantly lower LDH (498.11 ± 221.43 vs. 576.21 ± 239.84 U/L, *P*: 0.02), hematocrit (39.46 ± 6.03 vs. 40.81 ± 5.23 %, *P*: 0.04) MCV (84.47 ± 6.58 vs. 86.35 ± 5.35 fl, *P*: 0.008), and significantly higher lymphocytes (25.15 ± 11.29 vs. 21.93 ± 10.90 %, *P*: 0.01). Important parameters in different supplement categories are illustrated in Table 3. Recent supplementation was associated with lower BUN (31.31 ± 13.87 vs. 37.57 ± 19.77 mg/dL, *P*: 0.002) and higher 25(OH)D serum levels (28.13 ± 14.09 vs. 23.81 ± 13.55 ng/mL, *P*: 0.03). 25(OH)D serum level was significantly higher in those who had taken supplements in the past year (31.03 ± 13.20 vs. 22.29 ± 13.42; *P* < 0.001). Clinical and para-clinical parameters have been provided in Table 4. Compared to those who have not taken supplements recently, no parameter was significantly different in the supplemented group. After performing multiple variable adjustments, those with recent (0.70 ± 4.41 vs. 3.07 ± 9.96 days, *P* < 0.001) and long-term supplementation intake had fewer days of fever (0.74 ± 4.70 vs. 2.80 ± 9.46 days, *P* < 0.001). Furthermore, recent (91.83 ± 10.03 vs. 85.89 ± 21.06 %, *P* < 0.001) and long-term supplement consumption was associated with higher blood oxygen pressure after statistical adjustments (91.50 ± 11.20 vs. 86.77 ± 19.83 %, *P* < 0.001).

**Table 1:** Sociodemographic characteristics of patients with COVID-19 according to recent, long-term, and during hospitalization intakes.

	**Total**	**Recent supplement intake**		**Long-term supplement intake**	
		**No**	**Yes**	**No**	**Yes**
Sample size, *n*	300 (100%)	138 (46%)	162 (54%)	162 (54%)	138 (46%)
Age (years)	51.95± 15.34	54.72± 15.28^*^	49.59 ± 15.05	53.57 ± 15.37^*^	50.04 ± 15.14
**Gender**					
Men	164 (54.7%)	87 (63.1%)^*^	77 (47.5%)	101 (62.3%)^*^	63 (45.6%)
Women	136 (45.3%)	51 (36.9%)^*^	85 (52.5%)	61 (37.7%)^*^	75 (54.4%)
Number of family members	3.36± 1.29	3.46 ±1.47	3.29± 1.11	3.46 ± 1.39	3.25 ± 1.15
**Marital status**					
Single	143 (49.8%)	63 (47.4%)	80 (51.9%)	80 (52.2%)	63 (47%)
Married	143 (49.8%)	69 (51.9%)	74 (48.1%)	72 (47%)	71 (53%)
Divorced	1 (0.3%)	1 (0.8%)	0 (0%)	1 (0.6%)	0 (0%)
**Patient caregiver**					
Him/Herself	26 (8.7)	10 (7.2)	16 (9.9)	15 (9.3%)	11 (8%)
Spouse	50 (16.7%)	26 (20.3%)	24 (16.4%)	23 (14.2%)	27 (19.6%)
Children	31 (10.3%)	15 (11.7%)	16 (10.9%)	15 (9.3%)	16 (11.6%)
Spouse & children	192 (64%)	87 (67.9%)	105 (71.9%)	108 (66.7%)	84 (60.9%)
Nurse	1 (0.3%)	0 (0%)	1 (0.6%)	1 (0.6%)	0 (0%)
**Smoking status**					
Never smoker	260 (86.7%)	117 (84.7%)	143 (89.3%)	140 (86.4%)	120 (88.2%)
Former smoker	30 (10%)	15 (10.8%)	15 (9.3%)	16 (9.9%)	14 (10.3%)
Current smoker	8 (2.7%)	6 (4.3%)	2 (1.2%)	6 (3.7%)	2 (1.5%)
Smoking duration (years)	1.15 ± 5.40	1.29 ± 5.63	1.03 ± 5.21	1.04 ± 5.22	1.27 ± 5.62
**Smoking person in family**					
Yes	40 (13.3%)	17 (12.4%)	23 (14.2%)	20 (12.4%)	20 (14.4%)
No	259 (86.3%)	120 (87.5%)	139 (85.8%)	141 (87.6%)	118 (85.6%)
Alcohol history	11 (3.7%)	8 (5.8%)	3 (1.8%)	8 (4.9%)	3 (2.1%)
Drug abuse history	4 (1.3%)	3 (2.1%)	1 (0.6%)	3 (1.8%)	1 (0.7%)
**Life style habit**					
**Sleep change**					
Yes	152 (50.7%)	69 (50.7%)	83 (51.2%)	69 (42.9%)^*^	83 (60.6%)
No	146 (48.7%)	67 (49.3%)	79 (48.8%)	92 (57.1%)	54 (39.4%)
Sleep duration before illness	7.57± 2.07	7.49± 1.93	7.63 ± 2.18	7.52 ± 2.27	7.62 ± 1.82
Sleep duration after illness	6.86 ± 2.26	6.69 ±2.46	7 ±2.07	6.75 ± 2.22	6.98 ± 2.31
Taking sleeping pills before illness	8 (2.7%)	5 (3.6%)	3 (1.8%)	3 (1.8%)	5 (3.6%)
**Facing a stressful event in the last 6 months**					
Yes	125 (4.7%)	48 (35%) ^*^	77 (47.5%)	52 (32.2%)^*^	73 (52.8%)
No	174 (58%)	89 (65%) ^*^	85 (52.4%)	109 (67.8%)^*^	65 (47.2%)
**Physiologic condition**					
**Breast feeding**					
Yes	3 (1%)	2 (1.4%)	1 (0.6%)	1 (0.6%)	2 (1.4%)
No	297 (99%)	136 (98.6%)	161 (99.4%)	161 (99.4%)	136 (98.6%)
**Menopausal condition**					
Menopause	63 (21%)	26 (18.8%)	37 (22.8%)	33 (20.3%)	30 (21.7%)
Non-menopausal	237 (79%)	112 (81.2%)	125 (77.2%)	129 (79.7%)	108 (78.7%)
**Responder to the questionnaire**					
Patient	1 (0.3%)	0 (0%) ^*^	1 (0.6%)	1 (0.6%)^*^	0 (0%)
Other person	299 (99.7%)	138 (100%) ^*^	161 (99.4%)	161 (99.4%)^*^	138 (100%)

*B.Sc., A Bachelor of Science; M.Sc., A Master of Science; PH.D, Doctor of Philosophy. Qualitative variables are reported as frequency (percent). Quantitative variables are reported as mean ± SD*.

*P-value is reported based on one-way ANOVA test*.

* *P-value is < 0.05 and considered as statistically significant*.

**Table 2:** Anthropometric and obesity characteristics of patients with COVID-19.

	**Total**	**Recent supplement intake**		**Long-term supplement intake**	
		**No**	**Yes**	**No**	**Yes**
Height (cm)	168 ± 9.33	168.68 ± 9.56	167.43 ± 9.12	168.68 ± 9.37	167.22 ± 9.26
**Weight (kg)**					
Weight before illness (kg)	81.84 ± 16.34	81.88 ± 17.42	81.80 ± 15.41	82.24 ± 17.23	81.37 ± 15.27
Weight after illness (kg)	77.75 ± 15.88	77.23 ± 16.58	78.19 ± 15.30	77.94 ± 16.60	77.52 ± 15.05
**Sudden weight change in the last 6 months**					
Yes	47 (15.7%)	21 (15.4%)	26 (16%)	22 (13.7%)	25 (18.1%)
No	251 (84.3%)	115 (84.6%)	136 (84%)	138 (86.3%)	113 (81.9%)
**Sudden weight change in the past year**					
Yes	110 (36.7%)	58 (42.3%)	52 (32%)	72 (44.7%) ^*^	38 (27.5%)
No	189 (63%)	79 (57.7%)	110 (68%)	89 (55.3%)	100 (72.5%)
**Obesity**					
Yes	15 (5%)	3 (2.1%) ^*^	12 (7.4%)	7 (4.3%)	8 (5.7%)
No	285 (95%)	135 (97.9%)	150 (92.6%)	155 (95.7%)	130 (94.3%)
BMI (kg/m^2^)	27.48 ± 5.10	27.07 ± 5.25	27.83 ± 4.95	27.34 ± 5.44	27.65 ± 4.68

*BMI, body mass index. Qualitative variables are reported as frequency (percent). Quantitative variables are reported as mean ± SD*.

*P-value is reported based on one-way ANOVA test*.

* *P-value is < 0.05 and considered as statistically significant*.

**Table 3:** Important parameters in patients with COVID-19 in different supplement intake categories.

	**Total**	**Recent supplement intake**		**Long-term supplement intake**	
		**No**	**Yes**	**No**	**Yes**
BUN (mg/dL)	34.18± 17.09	37.57 ± 19.77^*^	31.31 ± 13.87	35.28 ±17.02	32.89 ± 17.15
Adjusted	34.44 ± 17.66	38.72 ± 20.35^*^	30.58 ± 13.88	35.68 ± 16.94^*^	33.06 ± 18.46
Cr (mg/dL)	1.14 ± 0.65	1.22 ± 0.89	1.07 ± 0.32	1.14 ± 0.62	1.14 ± 0.69
Adjusted	1.17 ± 0.72	1.25 ± 0.96^*^	1.11 ± 0.39	1.11 ± 0.42^*^	1.24 ± 0.95
Alb (g/dL)	4.17 ± 0.4	4.12 ± 0.42	4.21 ± 0.38	4.16 ± 0.39	4.18 ± 0.40
Adjusted	4.18 ± 0.38	4.14 ± 0.39^*^	4.22 ± 0.37	4.17 ± 0.37^*^	4.19 ± 0.40
Ferritin (ng/ml)	307.8 ± 270.34	299.20 ± 250.52	314.62 ± 285.97	320.16 ± 267.26	293.09 ± 274.64
Adjusted	299.03 ± 255.13	276.36 ± 244.83	319.44 ±264.15	315.86 ± 277.64	280.33 ± 228.31
Vitamin D (ng/mL)	26.18 ± 13.99	23.81 ± 13.55 ^*^	28.13 ± 14.09	22.29 ± 13.42 ^*^	31.03 ± 13.20
Adjusted	26.77 ± 14.47	23.73 ± 13.62	29.51 ± 14.77	22.25 ± 13.84	31.79 ± 13.57
CRP (mg/L)	19.91 ± 32.61	18.64 ± 28.53	20.97 ± 35.73	20.63 ± 36.94	19.06 ± 26.76
Adjusted	12.78 ± 21.21	15.13 ± 24.30^*^	10.66 ± 17.90	13.79 ± 22.83^*^	11.66 ± 19.39
D-Dimer (ng/mL)	247.21 ± 484.87	301.16 ± 574.74	206.92 ± 404.29	239.90 ± 407.58	255.92 ± 566.641
Adjusted	271.35 ± 577.86	352.83 ± 683.55	209.87 ± 481.04	256.19 ± 494.75	287.53 ± 660.47
Hb (g/dL)	13.81 ± 1.96	13.84 ± 2.08	13.78 ± 1.85	13.95 ± 1.97	13.63 ± 1.94
Adjusted	14.05 ± 1.92	14.05 ± 1.97^*^	14.06 ± 1.88	14.24 ± 1.84^*^	13.85 ± 2.00

*BUN, blood urea nitrogen; Cr, creatinine; Alb, albumin; CRP, C-reactive protein; Hb, hemoglobin. Qualitative variables are reported as frequency (percent). Quantitative variables are reported as mean ± SD. P-value is reported based on one-way ANOVA test*.

* *P-value is < 0.05 and considered as statistically significant*.

*Data are presented as x ± s. Model 1: The P-values were controlled by inpatient, gender, age, history and current onset of diabetes, hypertension, hyperlipidemia, ischemic heart disease, cerebrovascular accident, lung disease, kidney failure, psychiatry disease, obesity, immune deficiency disease, cardiovascular disease, transplant, chronic kidney disease, coronary artery disease, hypothyroid, asthma, congestive heart failure, osteoarthritis, Alzheimer's disease, rheumatoid arthritis, chronic obstructive pulmonary disease, end-stage renal disease, coronary heart disease, Parkinson's, hepatitis B, deep vein thrombosis, meningitis and taking hydroxychloroquine, azithromycin, heparin, oseltamivir, Kaletra, ribavirin, favipiravir, remdesivir, atazanavir, intravenous immune globulin, ivermectin, anticoagulants, psychotropic drugs body mass index, hemoglobin, nutrition overall score, existence of respiratory support, fever, blood oxygen level, partial pressure of carbon dioxide, albumin, vitamin D, and c-reactive protein which were included as covariates. P-value is reported based on one-way ANOVA test*.

* *P-value is < 0.05 and considered as statistically significant*.

**Table 4:** Clinical and paraclinical parameters in patients with COVID-19 in different supplement intake categories.

	**Total**	**Recent supplement intake**		**Long-term supplement intake**	
		**No**	**Yes**	**No**	**Yes**
Systolic BP (mmHg)	119.51 ± 15.82	121.19 ±15.61	117.75 ± 15.99	120.22 ± 16.41	118.62 ± 15.16
Adjusted	118.33 ± 15.20	121.94 ± 13.84	113.93 ± 15.86	118.88 ± 14.16	117.71 ± 16.54
Diastolic BP (mmHg)	69.82 ± 19.81	72.44 ± 17.67	67.31 ± 21.54	69.54 ± 21.39	70.17 ± 17.93
Adjusted	65.90 ± 23.70	73.15 ± 17.43	58.65 ± 27.08	66.44 ± 25.37	65.32 ± 22.27
Headache	60 (20%)	25 (18.1%)	35 (21.6%)	27 (19.6%)	33 (29.5%)
Adjusted	-	-	-	-	-
Fever	28 (9.3%)	16 (11.5%)	12 (7.4%)	17 (12.3%)	11 (9.8%)
Adjusted	-	-	-	-	-
Duration of fever (days)	1.67 ± 7.3	2.35 ± 8.75	1.05 ± 5.63	2.64 ± 9.21 *	0.49 ± 3.54
Adjusted	1.83 ± 7.65	3.07 ± 9.96*	0.70 ± 4.41	2.80 ± 9.46*	0.74 ± 4.70
Respiratory rate (breaths/minute)	19.3 ± 9.87	18.31 ± 5.03	20.21 ± 12.74	19.51 ± 13.05	19.05 ± 2.97
Adjusted	18.54 ± 4.52	18.31 ± 5.91*	18.75 ± 2.78	18.21 ± 5.43*	18.91 ± 3.24
Spo2 (%)	89.52 ± 15.24	87.98 ± 18.10	90.95 ± 11.91	87.44 ± 18.71*	92.09 ± 8.78
Adjusted	89.01 ± 16.43	85.89 ± 21.06*	91.83 ± 10.03	86.77 ± 19.83*	91.50 ± 11.20
Ventilator					
Yes	105 (35%)	58 (48.3%)	57 (43.8%)	68 (49.3%)	47 (42%)
Adjusted	-	-	-	-	-
No	135 (45%)	62 (51.7%)	73 (56.2%)	70 (50.7%)	65 (58%)
Adjusted	-	-	-	-	-
Duration of ventilator (days)	2.69 ± 4.12	2.38 ± 3.71	2.98 ± 4.47	2.78 ± 4.12	2.58 ± 4.14
Adjusted	2.61 ± 3.80	2.50 ± 3.75*	2.71 ± 3.86	2.56 ± 3.88*	2.66 ± 3.72
Invasive respiratory support	7 (2.3%)	5 (3.6%)	2 (1.2%)	7 (5.1%)*	0 (0%)
Adjusted	-	-	-	-	-
Non-invasive respiratory support	104 (34.6%)	50 (36.2%)	54 (33.3%)	58 (42%)	46 (41.1%)
Adjusted	-	-	-	-	-
Ocular congestion	8 (2.7%)	2 (1.4%)	6 (3.7%)	3 (2.2%)	5 (4.5%)
Adjusted	-	-	-	-	-
Pulse rate (beats/minute)	81.91 ± 21.85	80.15 ± 24.37	83.53 ± 19.19	79.79 ± 26.37	84.51 ± 14.17
Adjusted	81.98 ± 21.28	78.98 ± 25.59*	84.68 ± 16.20	81.11 ± 25.34*	82.94 ± 15.76
Sinusoidal heart rhythm	217 (72.3%)	99 (71.7%)	118 (72.8%)	127 (92.7%)	102 (91.9%)
Adjusted	-	-	-	-	-
Acute failure syndrome	5 (1.7%)	2 (1.4%)	3 (1.8%)	3 (2.2%)	2 (1.8%)
Adjusted	-	-	-	-	-

SBP, systolic blood pressure; DBP, diastolic blood pressure; Spo2, saturation of peripheral oxygen. Qualitative variables are reported as frequency (percent). Quantitative variables are reported as mean ± SD. P-value is reported based on one-way ANOVA test. *P-value is <0.05 and considered as statistically significant.

Data are presented as $\bar{x}$ ± s. Model 1: The P-values were controlled by inpatient, gender, age, history and current onset of diabetes, hypertension, hyperlipidemia, ischemic heart disease, cerebrovascular accident, lung disease, kidney failure, psychiatry disease, obesity, immune deficiency disease, cardiovascular disease, transplant, chronic kidney disease, coronary artery disease, hypothyroid, asthma, congestive heart failure, osteoarthritis, Alzheimer's disease, rheumatoid arthritis, chronic obstructive pulmonary disease, end-stage renal disease, coronary heart disease, Parkinson's, hepatitis B, deep vein thrombosis, meningitis and taking hydroxychloroquine, azithromycin, heparin, oseltamivir, Kaletra, ribavirin, favipiravir, remdesivir, atazanavir, intravenous immune globulin, ivermectin, anticoagulants, psychotropic drugs body mass index, hemoglobin, nutrition overall score, existence of respiratory support, fever, blood oxygen level, partial pressure of carbon dioxide, albumin, vitamin D, and c-reactive protein which were included as covariates. P-value is reported based on one-way ANOVA test. *P-value is < 0.05 and considered as statistically significant.

The length of hospitalization after gender, age, history and the current onset of diseases and history and current medications adjustment are presented in model 1 (Table 5 and Figure 1). The *P*-values were controlled by body mass index, hemoglobin and nutrition overall score, which were included as covariates additionally in model 2 (Table 5 and Figure 1). In model 3, *P*-values were controlled by the existence of respiratory support, fever and blood oxygen level, partial pressure of carbon dioxide, albumin, 25(OH)D, c-reactive protein serum levels, in addition to the above variables which were included as covariates. Although, those who recently supplemented had slightly fewer hospital stays, the length of hospitalization was practically the same (6.36 ± 3.32 vs. 6.71± 4.33 days, *P*: 0.004). Similarly, People who took supplements in the past year also had similar hospitalization lengths (6.29 ± 4.13 vs. 6.74± 3.55 days, *P*: 0.004) (Table 5 and Figure 1).

**Table 5:** Length of hospitalization in different supplement intake categories.

	**Total**	**Recent supplement intake**		**Total**	**Long-term supplement intake**	
		**Yes**	**No**		**Yes**	**No**
Crude	6.93 ± 4.99	6.99 ± 4.79	6.85 ± 5.22	6.93 ± 4.99	6.94 ± 5.12	6.91 ± 4.88
Model 1	6.42 ± 4.52	6.54± 4.20	6.30 ± 4.84	6.42 ± 4.52	6.43 ± 4.48	6.41 ± 4.57
Model 2	6.67 ± 4.57	6.68 ± 4.23	6.67 ± 4.91	6.67 ± 4.57	6.68 ± 4.52	6.67 ± 4.62
Model 3	6.53 ± 3.82	6.36 ± 3.32^*^	6.71 ± 4.33	6.53 ± 3.82	6.29 ± 4.13^*^	6.74 ± 3.55

*Data are presented as x̄ ± s. Model 1: The P-values were controlled by gender, age, history and current onset of diabetes, hypertension, hyperlipidemia, ischemic heart disease, cerebrovascular accident, lung disease, kidney failure, psychiatry disease, obesity, immune deficiency disease, cardiovascular disease, transplant, chronic kidney disease, coronary artery disease, hypothyroid, asthma, congestive heart failure, osteoarthritis, Alzheimer's disease, rheumatoid arthritis, chronic obstructive pulmonary disease, end-stage renal disease, coronary heart disease, Parkinson's, hepatitis B, deep vein thrombosis, meningitis and taking hydroxychloroquine, azithromycin, heparin, oseltamivir, Kaletra, ribavirin, favipiravir, remdesivir, atazanavir, intravenous immune globulin, ivermectin, anticoagulants, and psychotropic drugs which were included as covariates. Model 2: The P-values were controlled by body mass index, hemoglobin, and nutrition overall score which were included as covariates additionally. Model 3: The P-values were controlled by existence of respiratory support, fever and blood oxygen level, partial pressure of carbon dioxide, albumin, vitamin D, and c-reactive protein in addition to the above variables which were included as covariates. P-value is reported based on a one-way ANOVA test*.

* *P-value is < 0.05 and considered as statistically significant*.

## Discussion

In this cross-sectional study, we did not find a significant inverse relationship between supplementation in the last 2 months and the length of hospital stay among Iranian patients with COVID-19. Similarly, people who received supplements in the past year had similar hospitalization duration. Recent supplementation was significantly associated with lower BUN and higher serum 25(OH)D. Furthermore, 25(OH)D serum levels were significantly higher in those who had long-term supplementation during the past year. Moreover, recent supplementation was slightly associated with lower blood creatinine levels. To the best of our knowledge, this is the first study to completely examine the association of recent and long-term consumption of various supplements with different laboratory indicators and the length of hospital stay in patients with COVID-19.

The results of our study showed that people who took supplements modulating the immune system during the year before admission needed significantly less invasive support. This result was consistent with a cohort study that showed that the combination of vitamin D, magnesium, and B12 intake was associated with a reduced need for respiratory support and (or) hospitalization in the ICU (37). The possible mechanism of vitamin D is that it has an effect on NF-κB and reduces pro-inflammatory cytokines and reduces the risk of cytokine storm (38). Magnesium has a positive effect by acting as a cofactor in the synthesis and activation of vitamin D (39). Vitamin B12 also has a positive effect on the gut microbiota and promotes the innate and adaptive immunity of COVID-19 patients (40). Moreover, our study showed that adjuvant supplementation was significantly associated with lower BUN during the 2 months prior to admission. Our results were consistent with the findings of a clinical trial study that showed the effect of omega-3s on BUN reduction in the short term. The mechanism of action of omega-3 is probably by preventing the binding of the SARS-CoV-2 virus to the human ACE2 receptor and activating the host proteases TMPRSS2 and cathepsin L (41). Also, the results of our study were in line with the clinical trial study performed on healthy individuals, which showed a decrease in the ratio of BUN to creatinine after vitamin D supplementation (42). The possible mechanism of this favorable effect of vitamin D supplementation is an alteration in the composition of the microbiota gut, especially increasing Bacteroidetes to Firmicutes ratio (42). Our study findings showed a significant decrease in blood creatinine. This finding was consistent with the systematic review and meta-analysis findings on the use of probiotics and synbiotics that indicated a decrease in blood creatinine levels (43). We observed a significant reduction in CRP, which confirmed the findings of a clinical trial study that showed a significant reduction in inflammatory outcomes following supplementation with vitamins A, B, C, D, and E (44). Our study findings showed a significant decrease in blood LDH levels in supplemented groups. Previous evidence suggests that vitamin D supplementation significantly reduces LDH and CRP serum levels (45). Previous observational studies have mainly focused on the association of specific supplement intake rather than various supplements in COVID-19. A population-based study investigated the association of calcitriol supplementation with mortality in CKD patients and reported a significant association between supplementation and reduction in risk of COVID-19 infection and mortality rate (46). This study has been performed on CKD patients, and it is difficult to extend the results of this study to the general population. Also, some variables may not be considered in the matching process, which is an inherent limitation of this type of study. Furthermore, the study examined only calcitriol, but our study investigated all supplements involved in the immune system (46). A cross-sectional study examined the association of vitamin D supplementation and its outcomes in 19 patients and approached the significance of higher mortality rates (47). However, two of the three groups of participants in the study were either patients with Parkinson's or Parkinson's caregivers, which made it difficult to extrapolate these results to the general population (47). The study reviewed vitamin D supplementation in the last 3 months, but our study provides data on both recent (2 months ago) and long-term (1 year) intakes of all supplements involved in the immune system (47). Our findings from this study were in line with a clinical trial study, which showed that supplementation did not affect the number of days of hospitalization of COVID-19 patients (48, 49). In contrast, a clinical study demonstrated a favorable association between supplementation and hospital length of stay (50). Naturally, the results obtained from taking a particular supplement may differ from taking different types of supplements and their relationship to the length of hospital stay. Previous studies have examined the association of adjuvant supplementation with clinical symptoms. For instance, recently, a clinical trial showed that high doses of intravenous vitamin C significantly increased the percentage of oxygen saturation and decreased the respiratory rate and the percentage of lung involvement (48). The beneficial effects of vitamin C supplementation are exerted by correcting the disease-induced deficiency, reducing inflammation, increasing interferon production, and supporting the anti-inflammatory action of glucocorticosteroids in acute respiratory infections and critical COVID-19 patients (9). However, some studies did not find a significant effect of vitamin C supplementation on the duration of symptoms and length of hospital stay (51–53). Furthermore, several studies have investigated the association between vitamin D supplementation and clinical symptoms. Vitamin D supplementation improved blood oxygen levels, reduced oxygen requirement, reduced hospital length, and reduced mortality (33). A clinical trial study showed that following vitamin D supplementation, the ratio of arterial oxygen saturation to the inspired fraction of oxygen improved further (54). The vitamin D group also had a shorter average length of stay, less need for ICU transfers, and fewer deaths and readmissions (54). In addition, high-dose vitamin D supplementation normalized serum 25-OH vitamin D levels and was associated with shorter hospital stays, fewer oxygen requirements, and reduced inflammatory markers (33). Although vitamin D's exact mechanism of action in reducing the risk of infection is yet to be determined, the existing mechanisms are classified into three groups: physical barrier, natural cellular immunity, and adaptive immunity. Vitamin D can play a role in maintaining the physical barrier by improving tight junctions, gap junctions, and junction adhesions (55). Moreover, vitamin D induces antimicrobial peptides, such as human cathelicidin, LL-37, and defensins, thereby enhancing innate cellular immunity (56–58). Finally, vitamin D reduces cytokine storms, which results in enhanced cellular immunity (38). However, findings in this regard are inconsistent and some studies have shown that vitamin D supplementation has no effect on ventilator requirement, length of hospital stay, or mortality (59–62). Several studies have examined the association between zinc and clinical parameters. The effect of zinc supplementation on improving survival rate and reducing the length of hospital stay has been shown (63). Similarly, findings from a meta-analysis of 28 clinical trials in patients unlikely to be zinc deficient showed that zinc supplementation orally or intranasal reduced the symptoms of respiratory tract infections and improved the symptoms earlier (64). Likewise, zinc sulfate supplementation, as adjunctive therapy in critically ill patients, reduces 30-day mortality and has a potentially beneficial effect on kidney health (63). Zinc mediates mentioned desired effects by enhancing the anti-infective properties of basophils, eosinophils, and neutrophils (65). Moreover, according to *in vitro* research, it may inhibit RNA polymer, which needs further study (66, 67).

Various vitamins are involved in different stages of the immune response by the following possible mechanisms: Vitamin A plays a key role in boosting the function of innate immune cells, such as neutrophils, macrophages, NK cells, and boosting the antibody response (68, 69). Vitamins B6 and B12 also have a positive effect on the adaptive immune response by increasing the number of lymphocytes and enhancing the maturation of lymphocytes (70, 71). Vitamin C stimulates phagocytes and T lymphocytes and protects them against oxidative stress (68, 72). Studies have also shown that vitamins A, B, C, and E prevent cell tissue damage from the virus, and a deficiency in these vitamins can cause NK cells to malfunction (73, 74).

The results of our study showed that people with higher education have significantly more vitamin and mineral supplement intakes in the last 2 months. This is in agreement with Poland cross-sectional study which found that vitamin and mineral supplements, including vitamin D and zinc, were significantly higher in people with a medical degree (75).

Possible confounding variables were adjusted in three phases. One of the factors that may affect the laboratory indices and duration of hospitalization is medication. We examined the interaction of drugs with supplements in such a way that in a separate model, we performed statistical adjustments. If the effect of the interaction of drugs with supplements was significant, then the effect would disappear. In model 1, where the variables of gender, age, history and the current onset of diseases, and history and current medications were adjusted, no significant relationship was observed between supplementation and length of hospital stay. Furthermore, in model 2, no significant association was found following the adjustment of the covariates body mass index, hemoglobin, and nutrition overall score, additionally. Finally, after performing multiple variable adjustments in model 3, we could not find a practical significant favorable relationship between recent and long-term use of supplements with the length of hospital stay in COVID-19 patients. As we know, the nutritional status of the hospitalized person has a significant relationship with the duration of the patient's hospitalization; however, in our study, supplementation has not reduced the number of hospitalization days by improving the nutritional status. Overall, further studies appear to be needed to examine the association of recent and long-term adjuvant supplementation with the length of hospital stay in COVID-19 patients.

The main strength of this study was that it thoroughly investigated the relationship between the intakes of various immune-modulating supplements instead of one particular supplement with the laboratory indices of COVID-19 and the length of hospital stay. Another strength of this study is the complete study of the relationship between the use of supplements in short term and long term with various laboratory indicators of COVID-19 patients. In this cross-sectional study, some limitations should be considered in interpreting our results. According to the cross-sectional design of our study, the cause and effect relationship cannot be deduced from this study. However, it should be noted that a detailed analysis of the findings of the cross-sectional study can be used as a first step to determine the relationship between supplementation and length of hospital stay. Another limitation of this study was that it was conducted in a specific hospital. However, this limitation has been modified due to appropriate random selection and a sample size of 300 people. Furthermore, each questionnaire has its own biases that should be considered. For instance, due to the retrospective nature of our questionnaires and reliance on patients' memory, in some cases, the use of some supplements in the last 2 months and during the year before hospitalization may not be reported accurately. However, as we studied the intake of a large number of supplements, the effect of this error on our results is minimized. Similar studies may show different results due to the time-consuming process of our study and the rapid emergence of new strains of the COVID-19 virus.

## Conclusion

In conclusion, although practically the length of hospital stay was the same in both groups of supplement consumers and others, immune-boosting supplements were associated with improved several laboratory indices. The COVID-19 pandemic constantly raises the question of whether we need to take immune-boosting supplements. Another ambiguity is whether people who take immune-boosting supplements are less likely to develop the disease, which in our study 54% and 46% of COVID-19 patients had used the supplements in the last 2 months and year, respectively. Apart from the possibility of contracting the disease, there is another question whether patients with a history of supplementation are in a better condition in terms of complications and symptoms, length of hospital stay, laboratory, and paraclinical parameters, which this study can answer all these questions.

However, due to the cross-sectional nature of our study which cannot reveal causal relationships and given that new strains of COVID-19 are developing constantly, further longitudinal studies, concentrating on different types of supplements, seem to be essential.

## Data Availability Statement

The original contributions presented in the study are included in the article/Supplementary Materials, further inquiries can be directed to the corresponding author/s.

## Ethics Statement

The studies involving human participants were reviewed and approved by Tehran University of Medical Sciences. The patients/participants provided their written informed consent to participate in this study.

## Author Contributions

LA provided the idea for the study and supervised the study. LA, RH, and ZS designed the study settings. ZS drafted the proposal for this project and contributed to preparing the questionnaires. RH contributed to sampling, data collection, completing all the questionnaires related to this research, extracting data from the medical records, and entering the data into the software. LA and MM performed the statistical analysis. MM drafted the article and wrote the manuscript. All authors read and approved the final manuscript.

## Funding

This study was supported by the Tehran University of Medical Sciences (grant number: 99-1-212-47266).

## Conflict of Interest

The authors declare that the research was conducted in the absence of any commercial or financial relationships that could be construed as a potential conflict of interest.

## Publisher's Note

All claims expressed in this article are solely those of the authors and do not necessarily represent those of their affiliated organizations, or those of the publisher, the editors and the reviewers. Any product that may be evaluated in this article, or claim that may be made by its manufacturer, is not guaranteed or endorsed by the publisher.

## References

[1] Kumar A Kubota Y Chernov M Kasuya H. Potential role of zinc supplementation in prophylaxis and treatment of COVID-19. Medical Hypotheses. (2020) 144:109848. 10.1016/j.mehy.2020.109848 32512490 PMC7247509

[2] worldometers. COVID-19 CORONAVIRUS PANDEMIC worldometers: worldometers (2021), 12:52 GMT. Available online at: https://www.worldometers.info/coronavirus/ (accessed November 9, 2021).

[3] Centers for Disease Control and Prevention. When You've Been Fully Vaccinated October 15, 2021. Available online at: https://www.cdc.gov/coronavirus/2019-ncov/vaccines/fully-vaccinated_archived.html

[4] Lange KW Nakamura Y. Movement and nutrition in COVID-19. Journal of Disease Prevention and Health Promotion. (2020) 4. 10.5283/mnhd.33

[5] Caccialanza R Laviano A Lobascio F Montagna E Bruno R Ludovisi S. Early nutritional supplementation in non-critically ill patients hospitalized for the 2019 novel coronavirus disease (COVID-19): rationale and feasibility of a shared pragmatic protocol. Nutrition. (2020) 74:110835. 10.1016/j.nut.2020.110835 32280058 PMC7194616

[6] Carr AC Maggini S. Vitamin C and immune function. Nutrients. (2017) 9:1211. 10.3390/nu9111211 29099763 PMC5707683

[7] Gombart AF Pierre A Maggini S. A review of micronutrients and the immune system–working in harmony to reduce the risk of infection. Nutrients. (2020) 12:236. 10.3390/nu12010236 31963293 PMC7019735

[8] Grant WB Lahore H McDonnell SL Baggerly CA French CB Aliano JL. Evidence that vitamin D supplementation could reduce risk of influenza and COVID-19 infections and deaths. Nutrients. (2020) 12:988. 10.3390/nu12040988 32252338 PMC7231123

[9] Holford P Carr AC Jovic TH Ali SR Whitaker IS Marik PE. Vitamin C-an adjunctive therapy for respiratory infection, sepsis and COVID-19. Nutrients. (2020) 12:3760. 10.3390/nu12123760 33297491 PMC7762433

[10] Innes JK Calder PC. Marine omega-3 (N-3) fatty acids for cardiovascular health: an update for 2020. Int J Mol Sci. (2020) 21:1362. 10.3390/ijms21041362 32085487 PMC7072971

[11] Kieliszek M Lipinski B. Selenium supplementation in the prevention of coronavirus infections (COVID-19). Med Hypotheses. (2020) 143:109878. 10.1016/j.mehy.2020.109878 32464491 PMC7246001

[12] Martineau AR Jolliffe DA Hooper RL Greenberg L Aloia JF Bergman P. Vitamin D supplementation to prevent acute respiratory tract infections: systematic review and meta-analysis of individual participant data. BMJ. (2017) 356:6583. 10.1136/bmj.i6583 28202713 PMC5310969

[13] Rogero MM Leão MC Santana TM Pimentel M Carlini GCG da Silveira TFF. Potential benefits and risks of omega-3 fatty acids supplementation to patients with COVID-19. Free Radic Biol Med. (2020) 156:190–9. 10.1016/j.freeradbiomed.2020.07.005 32653511 PMC7350587

[14] Shakoor H Feehan J Al Dhaheri AS Ali HI Platat C Ismail LC. Immune-boosting role of vitamins D, C, E, zinc, selenium and omega-3 fatty acids: could they help against COVID-19? Maturitas. (2021) 143:1–9. 10.1016/j.maturitas.2020.08.003 33308613 PMC7415215

[15] Siuka D Pfeifer M Pinter B. Vitamin D supplementation during the COVID-19 pandemic. Mayo Clinic proceedings. (2020) 95:1804–5. 10.1016/j.mayocp.2020.05.036 32753156 PMC7275153

[16] Tang CF Ding H Jiao RQ Wu XX Kong LD. Possibility of magnesium supplementation for supportive treatment in patients with COVID-19. Eur J Pharmacol. (2020) 886:173546. 10.1016/j.ejphar.2020.173546 32931782 PMC7486870

[17] Alvarez-Sala J Alvarez-Mon M. Effect of immunomodulator AM3 on the exacerbations in patients with chronic bronchitis: a systematic review of controlled trials. Rev Clin Esp. (2004) 204:466–71. 10.1157/13065976 15388020

[18] Prieto A Reyes E Bernstein ED Martinez BN Monserrat J Izquierdo JL. Defective natural killer and phagocytic activities in chronic obstructive pulmonary disease are restored by glycophosphopeptical (Inmunoferon). Am J Resp Crit Care Med. (2001) 163:1578–83. 10.1164/ajrccm.163.7.2002015 11401877

[19] Fernández-Lázaro D Fernandez-Lazaro CI Mielgo-Ayuso J Adams DP Hernández JL González-Bernal J. Glycophosphopeptical AM3 food supplement: a potential adjuvant in the treatment and vaccination of SARS-CoV-2. Front Immunol. 2021;12. 10.3389/fimmu.2021.698672 34220861 PMC8248499

[20] Wessels I Rolles B Rink L. The potential impact of zinc supplementation on COVID-19 pathogenesis. Front Immunol. (2020) 11:1712. 10.3389/fimmu.2020.01712 32754164 PMC7365891

[21] Ali N. Role of vitamin D in preventing of COVID-19 infection, progression and severity. J Infect Public Health. (2020) 13:1373–80. 10.1016/j.jiph.2020.06.021 32605780 PMC7305922

[22] Chaabouni M Feki W Chaabouni K Kammoun S. Vitamin D supplementation to prevent COVID-19 in patients with COPD: a research perspective. Adv Res Med. (2020) 88:364–5. 10.5603/ARM.a2020.0101 32869273

[23] Chang R Ng TB Sun WZ. Lactoferrin as potential preventative and adjunct treatment for COVID-19. Int J Antimicrob Agents. (2020) 56:106118. 10.1016/j.ijantimicag.2020.106118 32738305 PMC7390755

[24] Mansur JL Tajer C Mariani J Inserra F Ferder L Manucha W. Vitamin D high doses supplementation could represent a promising alternative to prevent or treat COVID-19 infection. Clinica e investigacion en arteriosclerosis: publicacion oficial de la Sociedad Espanola de Arteriosclerosis. (2020) 32:267–77. 10.1016/j.artere.2020.11.003 32718670 PMC7256522

[25] Xu Y Baylink DJ Chen CS Reeves ME Xiao J Lacy C. The importance of vitamin d metabolism as a potential prophylactic, immunoregulatory and neuroprotective treatment for COVID-19. J Transl Med. (2020) 18:322. 10.1186/s12967-020-02488-5 32847594 PMC7447609

[26] Abobaker A Alzwi A Alraied AHA. Overview of the possible role of vitamin C in management of COVID-19. Pharmacol Reports: PR. (2020) 72:1517–28. 10.1007/s43440-020-00176-1 33113146 PMC7592143

[27] Ateya AM Sabri NA. Zinc supplementation for males during COVID-19: Is it beneficial? Med Hypotheses. (2021) 146:110403. 10.1016/j.mehy.2020.110403 33246691 PMC7677674

[28] Cengiz M Borku Uysal B Ikitimur H Ozcan E Islamoglu MS Aktepe E. Effect of oral l-Glutamine supplementation on Covid-19 treatment. Clin Nutr Exp. (2020) 33:24–31. 10.1016/j.yclnex.2020.07.003 32835086 PMC7387270

[29] Dhar D Mohanty A. Gut microbiota and Covid-19- possible link and implications. Virus Res. (2020) 285:198018. 10.1016/j.virusres.2020.198018 32430279 PMC7217790

[30] Koshak AE Koshak EA Mobeireek AF Badawi MA Wali SO Malibary HM. Nigella sativa supplementation to treat symptomatic mild COVID-19: a structured summary of a protocol for a randomized, controlled, clinical trial. Trials. (2020) 21:703. 10.1186/s13063-020-04647-x 32771034 PMC7414256

[31] Miryan M Soleimani D Dehghani L Sohrabi K Khorvash F Bagherniya M. The effect of propolis supplementation on clinical symptoms in patients with coronavirus (COVID-19): a structured summary of a study protocol for a randomized controlled trial. Trials. (2020) 21:996. 10.1186/s13063-020-04934-7 33272309 PMC7713667

[32] Moghaddam A Heller RA Sun Q Seelig J Cherkezov A Seibert L. Selenium deficiency is associated with mortality risk from COVID-19. Nutrients. (2020) 12:2098. 10.3390/nu12072098 32708526 PMC7400921

[33] Ohaegbulam KC Swalih M Patel P Smith MA Perrin R. Vitamin D supplementation in COVID-19 patients: a clinical case series. Am J Ther. (2020) 27:e485–e90. 10.1097/MJT.0000000000001222 32804682 PMC7473790

[34] Weill P Plissonneau C Legrand P Rioux V Thibault R. May omega-3 fatty acid dietary supplementation help reduce severe complications in Covid-19 patients? Biochimie. (2020) 179:275–80. 10.1016/j.biochi.2020.09.003 32920170 PMC7481803

[35] Alexander J Tinkov A Strand TA Alehagen U Skalny A Aaseth J. Early nutritional interventions with zinc, selenium and vitamin d for raising anti-viral resistance against progressive COVID-19. Nutrients. (2020) 12:2358. 10.3390/nu12082358 32784601 PMC7468884

[36] Azar M Sarkisian E. Food composition table of Iran. National Nutrition and Food Science Research Institute of Shaheed Beheshti University. Tehran (1981).

[37] Tan CW Ho LP Kalimuddin S Cherng BP Teh YE Thien SY. Cohort study to evaluate the effect of vitamin D, magnesium, and vitamin B12 in combination on progression to severe outcomes in older patients with coronavirus (COVID-19). Nutrition. (2020) 79:111017. 10.1016/j.nut.2020.111017 33039952 PMC7832811

[38] Sharifi A Vahedi H Nedjat S Rafiei H Hosseinzadeh-Attar MJ. Effect of single-dose injection of vitamin D on immune cytokines in ulcerative colitis patients: a randomized placebo-controlled trial. APMIS. (2019) 127:681–7. 10.1111/apm.12982 31274211

[39] Dai Q Zhu X Manson JE Song Y Li X Franke AA. Magnesium status and supplementation influence vitamin D status and metabolism: results from a randomized trial. Am J Clin Nutr. (2018) 108:1258–69. 10.1093/ajcn/nqy274 30541089 PMC6693398

[40] Degnan PH Taga ME Goodman AL. Vitamin B12 as a modulator of gut microbial ecology. Cell Metab. (2014) 20:769–78. 10.1016/j.cmet.2014.10.002 25440056 PMC4260394

[41] Doaei S Gholami S Rastgoo S Gholamalizadeh M Bourbour F Bagheri SE. The effect of omega-3 fatty acid supplementation on clinical and biochemical parameters of critically ill patients with COVID-19: a randomized clinical trial. J Transl Med. (2021) 19:128. 10.1186/s12967-021-02795-5 33781275 PMC8006115

[42] Singh P Rawat A Alwakeel M Sharif E Al Khodor S. The potential role of vitamin D supplementation as a gut microbiota modifier in healthy individuals. Sci Rep. (2020) 10:1–14. 10.1038/s41598-020-77806-4 33303854 PMC7729960

[43] Abdollahi S Meshkini F Clark CC Heshmati J Soltani S. The effect of probiotics/synbiotics supplementation on renal and liver biomarkers in patients with type 2 diabetes: a systematic review and meta-analysis of randomized-controlled trials. Br J Nutri. (2021) 1–31. 10.1017/S0007114521003780 34544511

[44] Beigmohammadi MT Bitarafan S Hoseindokht A Abdollahi A Amoozadeh L Soltani D. The effect of supplementation with vitamins A, B, C, D, and E on disease severity and inflammatory responses in patients with COVID-19: a randomized clinical trial. Trials. (2021) 22:802. 10.1186/s13063-021-05795-4 34776002 PMC8590866

[45] Lakkireddy M Gadiga SG Malathi RD Karra ML Raju IS Chinapaka S. Impact of daily high dose oral vitamin D therapy on the inflammatory markers in patients with COVID 19 disease. Sci Rep. (2021) 11:10641. 10.1038/s41598-021-97181-y 34017029 PMC8138022

[46] Oristrell J Oliva JC Subirana I Casado E Domínguez D Toloba A. Association of calcitriol supplementation with reduced covid-19 mortality in patients with chronic kidney disease: a population-based study. Biomedicines. (2021) 9:509. 10.3390/biomedicines9050509 34063015 PMC8147982

[47] Cereda E Bogliolo L Lobascio F Barichella M Zecchinelli AL Pezzoli G. Vitamin D supplementation and outcomes in coronavirus disease 2019 (COVID-19) patients from the outbreak area of Lombardy, Italy. Nutrition. (2021) 82:111055. 10.1016/j.nut.2020.111055 33288411 PMC7657015

[48] Tehrani S Yadegarynia D Abrishami A Moradi H Gharaei B Rauofi M. An investigation into the effects of intravenous vitamin C on pulmonary CT findings and clinical outcomes of patients with COVID 19 pneumonia a randomized clinical trial. Urol. J 18:6863. 10.22037/uj.v18i.6863 34746999

[49] Murai IH Fernandes AL Sales LP Pinto AJ Goessler KF Duran CS. Effect of vitamin D3 supplementation vs. placebo on hospital length of stay in patients with severe COVID-19: a multicenter, double-blind, randomized controlled trial. medRxiv. (2020) 325:1053–60. 10.1101/2020.11.16.2023239733595634

[50] Sadeghpour A Alizadehasl A Kyavar M Sadeghi T Moludi J Gholizadeh F. Impact of vitamin C supplementation on post-cardiac surgery ICU and hospital length of stay. Anesth Pain Med. (2015) 5:e25337. 10.5812/aapm.25337 25789244 PMC4350190

[51] Thomas S Patel D Bittel B Wolski K Wang Q Kumar A. Effect of high-dose zinc and ascorbic acid supplementation vs. usual care on symptom length and reduction among ambulatory patients with SARS-CoV-2 infection: the COVID A to Z randomized clinical trial. JAMA Network Open. (2021) 4:e210369. 10.1001/jamanetworkopen.2021.0369 33576820 PMC7881357

[52] Rawat D Roy A Maitra S Gulati A Khanna P Baidya DK. Vitamin C and COVID-19 treatment: a systematic review and meta-analysis of randomized controlled trials. Diabetes & metabolic syndrome. Clin Res Rev. (2021):102324. 10.1016/j.dsx.2021.102324 34739908 PMC8552785

[53] JamaliMoghadamSiahkali S Zarezade B Koolaji S SeyedAlinaghi S Zendehdel A Tabarestani M. Safety and effectiveness of high-dose vitamin C in patients with COVID-19: a randomized open-label clinical trial. Eur J Med Res. (2021) 26:1–9. 10.1186/s40001-021-00490-1 33573699 PMC7877333

[54] Elamir YM Amir H Lim S Rana YP Lopez CG Feliciano NV. A randomized pilot study using 490 calcitriol in hospitalized COVID-19 patients. Bone. (2022) 154:116175. 10.1016/j.bone.2021.116175 34508882 PMC8425676

[55] Schwalfenberg GK. A review of the critical role of vitamin D in the functioning of the immune system and the clinical implications of vitamin D deficiency. Mol Nutr Food Res. (2011) 55:96–108. 10.1002/mnfr.201000174 20824663

[56] Liu PT Stenger S Li H Wenzel L Tan BH Krutzik SR. Toll-like receptor triggering of a vitamin D-mediated human antimicrobial response. Science. (2006) 311:1770–3. 10.1126/science.1123933 16497887

[57] Adams JS Ren S Liu PT Chun RF Lagishetty V Gombart AF. Vitamin d-directed Rheostatic regulation of monocyte antibacterial responses. J Immunol. (2009) 182:4289–95. 10.4049/jimmunol.0803736 19299728 PMC2683618

[58] Laaksi I. Vitamin D and respiratory infection in adults. Proceed Nutr Soc. (2012) 71:90–7. 10.1017/S0029665111003351 22115013

[59] da Rocha AP Atallah AN Aldrighi JM Pires ALR dos Santos Puga ME Pinto ACPN. Insufficient evidence for vitamin D use in COVID-19: a rapid systematic review. Int J Clin Pract. (2021) 75:e14649. 10.1111/ijcp.14649 34310814 PMC8420259

[60] Güven M Gültekin H. The effect of high-dose parenteral vitamin D3 on COVID-19-related in hospital mortality in critical COVID-19 patients during intensive care unit admission: an observational cohort study. Eur J Clin Nutr. (2021) 75:1383–8. 10.1038/s41430-021-00984-5 34302132 PMC8299443

[61] Rawat D Roy A Maitra S Shankar V Khanna P Baidya DK. Vitamin D supplementation and COVID-19 treatment: a systematic review and meta-analysis. Diabetes & metabolic syndrome. Clin Res Rev. (2021) 15:102189. 10.1016/j.dsx.2021.102189 34217144 PMC8236412

[62] Arroyo-Díaz JA Julve J Vlacho B Corcoy R Ponte P Román E. Previous vitamin D supplementation and morbidity and mortality outcomes in people hospitalized for COVID19: a cross-sectional study. Front Pub Health. (2021) 9:8347. 10.3389/fpubh.2021.758347 34631653 PMC8498099

[63] Al Sulaiman K Al Juhani O Al Shaya AI Kharbosh A Kensara R Al Guwairy A. Evaluation of zinc sulfate as an adjunctive therapy in COVID-19 critically Ill patients: a two center propensity-score matched study. (2021) 9:1–14. 10.21203/rs.3.rs-572942/v1 34663411 PMC8522856

[64] Hunter J Arentz S Goldenberg J Yang G Beardsley J Myers SP. Zinc for the prevention or treatment of acute viral respiratory tract infections in adults: a rapid systematic review and meta-analysis of randomised controlled trials. BMJ open. (2021) 11. 34728441 10.1136/bmjopen-2020-047474 PMC8578211

[65] Gammoh NZ Rink L. Zinc in infection and inflammation. Nutrients. (2017) 9:624. 10.3390/nu9060624 28629136 PMC5490603

[66] te Velthuis AJ van den Worm SH Sims AC Baric RS Snijder EJ van Hemert MJ. Zn(2+) inhibits coronavirus and arterivirus RNA polymerase activity in vitro and zinc ionophores block the replication of these viruses in cell culture. PLoS Pathogens. (2010) 6:e1001176. 10.1371/journal.ppat.1001176 21079686 PMC2973827

[67] Krenn BM Gaudernak E Holzer B Lanke K Van Kuppeveld FJ Seipelt J. Antiviral activity of the zinc ionophores pyrithione and hinokitiol against picornavirus infections. J Virol. (2009) 83:58. 10.1128/JVI.01543-08 18922875 PMC2612303

[68] Maggini S Beveridge S Sorbara P Senatore G. Feeding the immune system: the role of micronutrients in restoring resistance to infections. CAB Rev: Perspect Agri, Veter Sci, Nutri Nat Res. (2008) 3:1–21. 10.1079/PAVSNNR20083098

[69] Maggini S Wintergerst ES Beveridge S Hornig DH. Selected vitamins and trace elements support immune function by strengthening epithelial barriers and cellular and humoral immune responses. Br J Nutri. (2007) 98:S29–35. 10.1017/S0007114507832971 17922955

[70] Wishart K. Increased micronutrient requirements during physiologically demanding situations: review of the current evidence. Vitamin Miner. (2017) 6:1–16. 10.4172/2376-1318.1000166

[71] Cheng C Chang S-J Lee B Lin K Huang Y. Vitamin B 6 supplementation increases immune responses in critically ill patients. Eur J Clin Nutr. (2006) 60:1207–13. 10.1038/sj.ejcn.1602439 16670691

[72] Anderson R Oosthuizen R Maritz R Theron A Van Rensburg A. The effects of increasing weekly doses of ascorbate on certain cellular and humoral immune functions in normal volunteers. Am J Clin Nutr. (1980) 33:71–6. 10.1093/ajcn/33.1.71 7355784

[73] Hemilä H Chalker E. Vitamin C can shorten the length of stay in the ICU: a meta-analysis. Nutrients. (2019) 11:708. 10.3390/nu11040708 30934660 PMC6521194

[74] Erickson KL Medina EA Hubbard NE. Micronutrients and innate immunity. J Infect Dis. (2000) 182(Supplement_1):S5–S10 10.1086/315922 10944478

[75] Puścion-Jakubik A Bielecka J Grabia M Mielech A Markiewicz-Zukowska R Mielcarek K. Consumption of food supplements during the three COVID-19 waves in Poland—focus on Zinc and Vitamin D. Nutrients. (2021) 13:3361. 10.3390/nu13103361 34684363 PMC8538476

