# Supplementary material for: Association of Recent and Long-Term Supplement Intakes With Laboratory Indices in Patients With COVID-19 in Tehran, Iran, During 2020
Source: Front Nutr. 2022 Jun 6;9:834826. doi: 10.3389/fnut.2022.834826 (PMC9207418; doi:10.3389/fnut.2022.834826)
Supplement: Supplementary file 1 [file Table_1.docx]

**Table1. Sociodemographic characteristics of patients with COVID-19 according to recent, long term and during hospitalization intake**

|  | **Total** | **Recent supplement intake** | | **Long-term supplement intake** | |
| --- | --- | --- | --- | --- | --- |
|  |  | **No** | **Yes** | **No** | **Yes** |
| **Education level** |  |  |  |  |  |
| **Illiterate** | **58 (19.3%)** | **33 (23.9%)** | **25 (15.4%)** | **35 (21.6%)** | **23 (16.7%)** |
| **Elementary school** | **70 (23.3%)** | **34 (24.6%)** | **36 (22.2%)** | **39 (24.1%)** | **31 (22.5%)** |
| **Middle school** | **50 (16.7%)** | **28 (20.3%)** | **22 (13.6%)** | **30 (18.5%)** | **20 (14.5%)** |
| **Diploma** | **75 (25%)** | **26 (18.8%)** | **49 (30.2%)** | **39 (24.1%)** | **36 (26.1%)** |
| **B.Sc.** | **40 (13.3%)** | **15 (10.9%)** | **25 (15.4%)** | **17 (10.5%)** | **23 (16.7%)** |
| **M.Sc. and PH.D** | **7 (2.3%)** | **2 (1.4%)** | **5 (3.1%)** | **2 (1.2%)** | **5 (3.6%)** |
| **Type of residence** |  |  |  |  |  |
| **Urban** | **300 (100%)** | **138 (100%)** | **162 (100%)** | **162 (100%)** | **138 (100%)** |
| **Rural** | **0 (0%)** | **0 (0%)** | **0 (0%)** | **0 (0%)** | **0 (0%)** |
| **Place of residence** |  |  |  |  |  |
| **Tehran** | **276 (92%)** | **124 (89.8%)** | **152 (93.8%)** | **146 (90.1%)** | **130 (94.2%)** |
| **Other** | **24 (8%)** | **14 (10.2%)** | **10 (6.2%)** | **16 (9.9%)** | **8 (5.7%)** |
| **Travelling abroad** |  |  |  |  |  |
| **Yes** | **20 (6.7%)** | **8 (6.6%)** | **12 (9.2%)** | **7 (5%)** | **13 (11.6%)** |
| **No** | **230 (76.7%)** | **112 (93.4%)** | **118 (90.8%)** | **131 (95%)** | **99 (88.4%)** |
| **Household heading status** |  |  |  |  |  |
| **Head of household** | **145 (48.3%)** | **77 (57%) *** | **68 (42.7%)** | **90 (56.3%)*** | **55 (41%)** |
| **Self** | **29 (9.7%)** | **12 (8.8%) *** | **17 (10.6%)** | **15 (9.4%)*** | **14 (10.4%)** |
| **Under supervision** | **120 (40%)** | **46 (34%) *** | **74 (46.5%)** | **55 (34.4%)*** | **65 (48.5%)** |
| **Job classification** |  |  |  |  |  |
| **Unemployed** | **9 (3%)** | **4 (2.8%)** | **5 (3%)** | **7 (4.3%)** | **2 (1.4%)** |
| **Worker** | **14 (4.7%)** | **6 (4.3%)** | **8 (4.9%)** | **9 (5.6%)** | **5 (3.6%)** |
| **Freelance** | **64 (21.3%)** | **34 (24.6%)** | **30 (18.5%)** | **37 (22.8%)** | **27 (19.6%)** |
| **Retired** | **41 (13.7%)** | **26 (18.8%)** | **15 (9.2%)** | **26 (16%)** | **15 (10.9%)** |
| **housewife** | **112 (37.3%)** | **47 (34%)** | **65 (40.1%)** | **53 (32.7%)** | **59 (42.8%)** |
| **Employee** | **53 (17.7%)** | **19 (13.7%)** | **34 (20.9%)** | **27 (16.7%)** | **26 (18.8%)** |
| **Other** | **7 (2.3%)** | **2 (1.4%)** | **5 (3%)** | **3 (1.9%)** | **4 (2.9%)** |
| **Number of lactations** | **1.44 ± 2.23** | **1.42 ± 2.32** | **1.46 ± 2.17** | **1.38 ± 2.33** | **1.51 ± 2.12** |
| **Pregnant** |  |  |  |  |  |
| **Yes** | **1 (0.3%)** | **0 (0%)** | **1 (0.6%)** | **0 (0%)** | **1 (0.7%)** |
| **No** | **299 (99.7%)** | **138 (100%)** | **161 (99.4%)** | **162 (100%)** | **137 (99.3%)** |
| **Number of childbirths** | **1.79 ±2.35** | **1.66 ± 2.57** | **1.73 ± 2.39** | **1.64 ± 2.41** | **1.96 ± 2.26** |

B.Sc.: A Bachelor of Science; M.Sc.: A Master of Science; PH.D: Doctor of Philosophy. Qualitative variables is reported as frequency (percent). Quantitative variables is reported as mean ± SD.

P-value is reported based on one-way ANOVA test.*P-value is <0.05 and considered as statistically significant.

**Table 2. History of different diseases and diagnosis methods among patients with COVID-19**

|  | **Total** | **Recent supplement intake** | | **Long-term supplement intake** | |
| --- | --- | --- | --- | --- | --- |
|  |  | **No** | **Yes** | **No** | **Yes** |
| **Co-morbidities** |  |  |  |  |  |
| **Diabetes** | **33 (11%)** | **16 (11.5%)** | **17 (10.4%)** | **15 (9.2%)** | **18 (13%)** |
| **Hypertension** | **42 (14%)** | **23 (16.6%)** | **19 (11.7%)** | **23 (14.1%)** | **19 (13.7%)** |
| **Hyperlipidemia** | **9 (3%)** | **7 (5%)** | **2 (1.2%)** | **3 (1.8%)** | **6 (4.3%)** |
| **Ischemic Heart disease** | **10 (3.3%)** | **7 (5%)** | **3 (1.8%)** | **7 (4.3%)** | **3 (2.1%)** |
| **CHD** | **1 (0.3%)** | **1 (0.7%)** | **0 (0%)** | **1 (0.6%)** | **0 (0%)** |
| **CAD** | **0 (0%)** | **0 (0%)** | **0 (0%)** | **0 (0%)** | **0 (0%)** |
| **Hypothyroid** | **5 (1.7%)** | **2 (1.4%)** | **3 (1.8%)** | **2 (1.2%)** | **3 (2.1%)** |
| **Asthma** | **3 (1%)** | **0 (0%)** | **3 (1.8%)** | **0 (0%)** ***** | **3 (2.1%)** |
| **CHF** | **2 (0.7%)** | **1 (0.7%)** | **1 (0.6%)** | **2 (1.2%)** | **0 (0%)** |
| **Arthroses** | **1 (0.3%)** | **0 (0%)** | **1 (0.6%)** | **0 (0%)** | **1 (0.7%)** |
| **Alzheimer** | **2 (0.7%)** | **1 (0.7%)** | **1 (0.6%)** | **1 (0.6%)** | **1 (0.7%)** |
| **Heart valve replacement** | **1 (0.3%)** | **1 (0.7%)** | **0 (0%)** | **0 (0%)** | **1 (0.7%)** |
| **Rheumatoid arthritis** | **2 (0.7%)** | **1 (0.7%)** | **1 (0.6%)** | **0 (0%)** | **2 (1.4%)** |
| **COPD** | **1 (0.3%)** | **1 (0.7%)** | **0 (0%)** | **0 (0%)** | **1 (0.7%)** |
| **ESRD** | **1 (0.3%)** | **1 (0.7%)** | **0 (0%)** | **0 (0%)** | **1 (0.7%)** |
| **Dialysis history** | **1 (0.3%)** | **1 (0.7%)** | **0 (0%)** | **0 (0%)** | **1 (0.7%)** |
| **CABG** | **3 (1%)** | **1 (0.7%)** | **2 (1.2%)** | **2 (1.2%)** | **1 (0.7%)** |
| **Parkinson** | **1 (0.3%)** | **1 (0.7%)** | **0 (0%)** | **1 (0.6%)** | **0 (0%)** |
| **History of liver surgery** | **1 (0.3%)** | **0 (0%)** | **1 (0.6%)** | **1 (0.6%)** | **0 (0%)** |
| **Kidney failure** | **1 (0.3%)** | **1 (0.7%)** | **0 (0%)** | **0 (0%)** | **1 (0.7%)** |
| **HBV** | **1 (0.3%)** | **1 (0.7%)** | **0 (0%)** | **1 (0.6%)** | **0 (0%)** |
| **DVT** | **1 (0.3%)** | **1 (0.7%)** | **0 (0%)** | **1 (0.6%)** | **0 (0%)** |
| **Meningitis** | **1 (0.3%)** | **1 (0.7%)** | **0 (0%)** | **1 (0.6%)** | **0 (0%)** |
| **HIV** | **7 (2.3%)** | **5 (3.6%)** | **2 (1.2%)** | **6 (3.6%)** | **1 (0.7%)** |
| **CVA** | **2 (0.7%)** | **2 (1.4%)** | **0 (0%)** | **2 (1.2%)** | **0 (0%)** |
| **Chronic kidney disease** | **2 (0.7%)** | **2 (1.4%)** | **0 (0%)** | **1 (0.6%)** | **1 (0.7%)** |
| **Mental disease** | **17 (5.7%)** | **6 (4.3%)** | **11 (6.7%)** | **8 (4.9%)** | **9 (6.5%)** |
| **Obesity** | **15 (5%)** | **3 (2.1%)** ***** | **12 (7.4%)** | **7 (4.3%)** | **8 (5.7%)** |
| **Immune deficiency** | **5 (1.7%)** | **2 (1.4%)** | **3 (1.8%)** | **0 (0%)** ***** | **5 (3.6%)** |
| **Transplant** | **1 (0.3%)** | **1 (0.7%)** | **0 (0%)** | **0 (0%)** | **0 (0%)** |
| **COVID-19 Hospitalization** |  |  |  |  |  |
| **Yes** | **281 (93.7%)** | **128 (93.4%)** | **153 (95.6%)** | **151 (94.3%)** | **130 (94.8%)** |
| **No** | **16 (5.3%)** | **9 (6.6%)** | **7 (4.3%)** | **9 (5.7%)** | **7 (5.2%)** |
| **Place of hospitalization** |  |  |  |  |  |
| **Emergency** | **23 (7.7%)** | **10 (7.2%)** | **13 (8%)** | **11 (6.8%)** | **12 (8.7%)** |
| **Ward** | **260 (86.7%)** | **117 (84.7%)** | **143 (88.2%)** | **142 (87.7%)** | **118 (85.5%)** |
| **ICU** | **8 (2.7%)** | **6 (4.3%)** | **2 (1.2%)** | **5 (3.1%)** | **3 (2.2%)** |
| **ICU Ward** | **9 (3%)** | **5 (3.6%)** | **4 (2.4%)** | **4 (2.5%)** | **5 (3.6%)** |
| **Hospitalization duration (Days)** | **6.93 ± 4.99** | **6.85± 5.22** | **6.99 ± 4.79** | **6.91 ± 4.88** | **6.94 ± 5.12** |
| **Discharge condition** |  |  |  |  |  |
| **Discharge in good general condition** | **285 (95%)** | **129 (94.1%)** | **156 (96.2%)** | **154 (95.7%)** | **131 (94.9%)** |
| **Discharge with personal consent** | **11 (3.7%)** | **7 (5.1%)** | **4 (2.4%)** | **6 (3.7%)** | **5 (3.6%)** |
| **Others** | **3 (1%)** | **1 (0.7%)** | **2 (1.2%)** | **1 (0.6%)** | **2 (1.4%)** |
| **Method of diagnosis** |  |  |  |  |  |
| **Physician** | **7 (2.3%)** | **5 (3.7%)** | **2 (1.2%)** | **6 (3.8%)** | **1 (0.7%)** |
| **Covid-19 test** | **21 (7%)** | **9 (6.7%)** | **12 (7.4%)** | **10 (6.3%)** | **11 (8%)** |
| **Lung CT scan** | **189 (63%)** | **82 (61.1%)** | **107 (66%)** | **100 (63.3%)** | **89 (64.5%)** |
| **Physician & Lung CT scan** | **21 (7%)** | **8 (5.9%)** | **13 (8%)** | **11 (7%)** | **10 (7.2%)** |
| **Covid-19 test & Lung CT scan** | **50 (16.7%)** | **26 (19.4%)** | **24 (14.8%)** | **27 (17.1%)** | **23 (16.7%)** |
| **Covid-19 test & Lung CT scan & Physician** | **8 (2.7%)** | **4 (2.9%)** | **4 (2.4%)** | **4 (2.5%)** | **4 (2.9%)** |
| **Contact with an infected person in the last 6 months** |  |  |  |  |  |
| **Yes** | **133 (44.3%)** | **63 (45.9%)** | **70 (43.2%)** | **70 (43.4%)** | **63 (45.6%)** |
| **No** | **166 (55.3%)** | **74 (54.1%)** | **92 (56.8%)** | **91 (56.6%)** | **75 (54.4%)** |

CHD: Coronary heart disease; CAD: Coronary Artery Disease; CHF: Congestive heart failure; COPD: Chronic obstructive pulmonary disease ESRD: End-Stage Renal Disease; CABG: A coronary artery bypass graft; HBV: Hepatitis B; DVT: deep vein thrombosis; HIV: human immunodeficiency virus; CVA: cerebrovascular accident. Qualitative variables is reported as frequency (percent). Quantitative variables is reported as mean ± SD. P-value is reported based on one-way ANOVA test. *P-value is <0.05 and considered as statistically significant.

**Table 3. Dietary intake during hospitalization among patients with COVID-19 in different supplement categories intake**

|  | **Total** | **Recent supplement intake** | | **Long-term supplement intake** | |
| --- | --- | --- | --- | --- | --- |
|  |  | **No** | **Yes** | **No** | **Yes** |
| **The amount of breakfast intake in comparison with usual intake (ratio)** |  |  |  |  |  |
| **Nothing** | **55 (18.3%)** | **36 (27%)** ***** | **19 (11.7%)** | **32 (20.4%)** | **23 (16.7%)** |
| **¼** | **74 (24.7%)** | **25 (18.7%)** ***** | **49 (30.2%)** | **38 (24.2%)** | **36 (26.1%)** |
| **½** | **38 (12.7%)** | **13 (9.7%)** ***** | **25 (15.4%)** | **22 (14%)** | **16 (11.6%)** |
| **¾** | **18 (6%)** | **7 (5.2%)** ***** | **11 (6.7%)** | **8 (5.1%)** | **10 (7.2%)** |
| **Complete** | **110 (36.7%)** | **52 (39%)** ***** | **58 (35.8%)** | **57 (36.3%)** | **53 (38.4%)** |
| **The amount of breakfast snack intake in comparison with usual intake (ratio)** |  |  |  |  |  |
| **Nothing** | **57 (19%)** | **26 (19.5%)** | **31 (19.1%)** | **28 (17.8%)** | **29 (21%)** |
| **¼** | **36 (12%)** | **14 (10.5%)** | **22 (13.5%)** | **17 (10.8%)** | **19 (13.8%)** |
| **½** | **26 (8.7%)** | **11 (8.2%)** | **15 (9.2%)** | **13 (8.3%)** | **13 (9.4%)** |
| **¾** | **9 (3%)** | **3 (2.2%)** | **6 (3.7%)** | **5 (3.2%)** | **4 (2.9%)** |
| **Complete** | **167 (55.7%)** | **79 (59.3%)** | **88 (54.3%)** | **94 (59.9%)** | **73 (52.9%)** |
| **The amount of launch intake in comparison with usual intake (ratio)** |  |  |  |  |  |
| **Nothing** | **53 (17.7%)** | **32 (24%)** | **21 (12.9%)** | **30 (19.1%)** | **23 (16.7%)** |
| **¼** | **83 (27.7%)** | **33 (24.8%)** | **50 (30.8%)** | **45 (28.7%)** | **38 (27.5%)** |
| **½** | **42 (14%)** | **16 (12%)** | **26 (16%)** | **23 (14.6%)** | **19 (13.8%)** |
| **¾** | **33 (11%)** | **13 (9.7%)** | **20 (12.3%)** | **16 (10.2%)** | **17 (12.3%)** |
| **Complete** | **84 (28%)** | **39 (29.3%)** | **45 (27.7%)** | **43 (27.4%)** | **41 (29.7%)** |
| **The amount of evening snack intake in comparison with usual intake (ratio)** |  |  |  |  |  |
| **Nothing** | **57 (19%)** | **29 (21.8%)** | **28 (17.3%)** | **31 (19.9%)** | **26 (18.8%)** |
| **¼** | **43 (14.3%)** | **14 (10.5%)** | **29 (18%)** | **18 (11.5%)** | **25 (18.1%)** |
| **½** | **34 (11.3%)** | **14 (10.5%)** | **20 (12.4%)** | **14 (9%)** | **20 (14.5%)** |
| **¾** | **9 (3%)** | **4 (3%)** | **5 (3.1%)** | **5 (3.2%)** | **4 (2.9%)** |
| **Complete** | **151 (50.3%)** | **72 (54.1%)** | **79 (49%)** | **88 (56.4%)** | **63 (45.7%)** |
| **The amount of dinner intake in comparison with usual intake (ratio)** |  |  |  |  |  |
| **Nothing** | **53 (17.7%)** | **32 (24%)** ***** | **21 (13%)** | **28 (17.9%)** | **25 (18.1%)** |
| **¼** | **88 (29.3%)** | **31 (23.3%)** ***** | **57 (35.4%)** | **46 (29.5%)** | **42 (30.4%)** |
| **½** | **50 (16.7%)** | **20 (15%)** ***** | **30 (18.6%)** | **26 (16.7%)** | **24 (17.4%)** |
| **¾** | **28 (9.3%)** | **12 (9%)** ***** | **16 (9.9%)** | **16 (10.3%)** | **12 (8.7%)** |
| **Complete** | **75 (25%)** | **38 (28.5%)** | **37 (22.9%)** | **40 (25.6%)** | **35 (25.4%)** |
| **Medications** |  |  |  |  |  |
| **ACEI** | **9 (3%)** | **7 (5%)** | **2 (1.2%)** | **6 (4.3%)** | **3 (2.7%)** |
| **Hydroxychloroquine** | **241(80.3%)** | **109 (78.9%)** | **132 (81.4%)** | **123 (77.4%)** | **113 (84.3%)** |
| **Azithromycin** | **241 (80.3%)** | **109 (78.9%)** | **132 (81.4%)** | **131 (82.9%)** | **110 (82.1%)** |
| **Heparin** | **274 (91.3%)** | **126 (91.3%)** | **148 (91.3%)** | **148 (83.1%)** | **126 (84%)** |
| **Oseltamivir** | **121 (40.4%)** | **64 (46.3%)** ***** | **57 (35.1%)** | **68 (42.8%)** | **53 (39.6%)** |
| **Kaletra** | **120 (40%)** | **53 (38.4%)** | **67 (41.3%)** | **65 (40.9%)** | **55 (42%)** |
| **Ribavirine** | **10 (3.4%)** | **5 (3.6%)** | **5 (3%)** | **5 (3.4%)** | **5 (3.8%)** |
| **Favipiravir** | **41 (13.6%)** | **17 (12.3%)** | **24 (14.8%)** | **22 (13.6%)** | **19 (13.9%)** |
| **Remdesivir** | **22 (7.3%)** | **11 (7.9%)** | **11 (6.7%)** | **9 (5.6%)** | **13 (9.5%)** |
| **Atazanavir** | **55 (18.4%)** | **28 (20.2%)** | **27 (16.6%)** | **29 (17.9%)** | **26 (19%)** |
| **Ivermectin** | **6 (2%)** | **4 (2.8%)** | **2 (1.2%)** | **3 (1.2%)** | **3 (2.2%)** |

ACEI: Angiotensin-converting-enzyme inhibitor; SBP: systolic blood pressure; DBP: diastolic blood pressure. Qualitative variables is reported as frequency (percent). Quantitative variables is reported as mean ± SD.

P-value is reported based on one-way ANOVA test.

*P-value is <0.05 and considered as statistically significant.

**Table 4. Usual dietary intake among patients with COVID-19 in different supplement categories intake**

|  | **Total** | **Recent supplement intake** | | **Long-term supplement intake** | |
| --- | --- | --- | --- | --- | --- |
|  |  | **No** | **Yes** | **No** | **Yes** |
| **Following a special diet** |  |  |  |  |  |
| **Diabetic Diet** | **13 (4.3%)** | **5 (3.6%)** | **8 (4.9%)** | **7 (4.3%)** | **6 (4.3%)** |
| **Low fat Diet** | **8 (2.7%)** | **3 (2.2%)** | **5 (3.1%)** | **4 (2.5%)** | **4 (2.9%)** |
| **High protein Diet** | **2 (0.7%)** | **1 (0.7%)** | **1 (0.6%)** | **0 (0%)** | **2 (1.4%)** |
| **Low calorie Diet** | **3 (1%)** | **1 (0.7%)** | **2 (1.2%)** | **1 (0.6%)** | **2 (1.4%)** |
| **Ketogenic Diet** | **1 (0.3%)** | **1 (0.7%)** | **0 (0%)** | **1 (0.6%)** | **0 (0%)** |
| **High protein & Low fat Diet** | **1 (0.3%)** | **0 (0%)** | **1 (1.2%)** | **0 (0%)** | **1 (0.7%)** |
| **Low fat & Low calorie Diet** | **2 (0.7%)** | **2 (1.4%)** | **0 (0%)** | **1 (0.6%)** | **1 (0.7%)** |
| **Uremic Diet** | **1 (0.3%)** | **1 (0.7%)** | **0 (0%)** | **1 (0.6%)** | **0 (0%)** |
| **A diet rich in fruits and vegetables** | **222 (74%)** | **101 (74.3%)** | **121 (75.2%)** | **123 (77.4%)** | **99 (71.7%)** |
| **The amount of fruit consumed daily in the past year (serving)** |  |  |  |  |  |
| **0** | **3 (1%)** | **1 (0.7%)** | **2 (1.2%)** | **0 (0%)** | **3 (2.2%)** |
| **1** | **126 (42%)** | **57 (42.5%)** | **69 (42.9%)** | **68 (43%)** | **58 (42.3%)** |
| **2** | **86 (28.7%)** | **39 (29.1%)** | **47 (29.2%)** | **48 (30.4%)** | **38 (27.7%)** |
| **3 and more** | **80 (26.7%)** | **37 (27.6%)** | **43 (26.7%)** | **42 (26.6%)** | **38 (27.7%)** |
| **Consumption of carbonated beverages** |  |  |  |  |  |
| **Yes** | **192 (64%)** | **82 (60.2%)** | **110 (67.9%)** | **104 (65%)** | **88 (63.8%)** |
| **No** | **106 (35.3%)** | **54 (39.8%)** | **52 (32.1%)** | **56 (35%)** | **50 (36.2%)** |
| **Amount of carbonated beverages** |  |  |  |  |  |
| **No amount** | **101 (33.7%)** | **52 (38.5%)** | **49 (30.4%)** | **53 (33.5%)** ***** | **48 (34.8%)** |
| **Daily 1 to 3 (glasses)** | **19 (6.3%)** | **10 (7.4%)** | **9 (5.6%)** | **14 (8.9%)** | **5 (3.6%)** |
| **Daily >1 (bottle)** | **2 (0.7%)** | **0 (0%)** | **2 (1.2%)** | **0 (0%)** | **2 (1.4%)** |
| **Weekly 1 to 3 (glasses)** | **53 (17.7%)** | **22 (16.3%)** | **31 (19.3%)** | **22 (13.9%)** | **31 (22.5%)** |
| **Weekly 1 (bottle)** | **6 (2%)** | **4 (3%)** | **2 (1.2%)** | **5 (3.2%)** | **1 (0.7%)** |
| **Monthly 1 to 3 (glasses)** | **95 (31.7%)** | **39 (28.9%)** | **56 (34.8%)** | **56 (35.4%)** | **39 (28.3%)** |
| **Monthly 1 (bottle)** | **5 (1.7%)** | **2 (1.5%)** | **3 (1.9%)** | **3 (1.9%)** | **2 (1.4%)** |
| **Yearly** | **15 (5%)** | **6 (4.4%)** | **9 (5.6%)** | **5 (3.2%)** | **10 (7.2%)** |
| **Daily water consumption (glass)** |  |  |  |  |  |
| **1 to 4** | **141 (47%)** | **60 (46.2%)** | **81 (51.9%)** | **76 (49%)** | **65 (49.6%)** |
| **4 to 8** | **105 (35%)** | **46 (35.4%)** | **59 (37.8%)** | **57 (36.8%)** | **48 (36.6%)** |
| **More** | **40 (13.3%)** | **24 (18.5%)** | **16 (10.3%)** | **22 (14.2%)** | **18 (13.7%)** |
| **Type of bread consumed in last 6 months** |  |  |  |  |  |
| **Whole bread** | **57 (19%)** | **27 (19.9%)** | **30 (18.5%)** | **30 (18.8%)** | **27 (19.6%)** |
| **White bread** | **241 (80.3%)** | **109 (80.1%)** | **132 (81.5%)** | **130 (81.3%)** | **111 (80.4%)** |
| **The amount of bread consumed during last year (counts)** |  |  |  |  |  |
| **<1/2** | **31 (10.3%)** | **12 (8.8%)** | **19 (11.7%)** | **12 (7.5%)** | **19 (13.8%)** |
| **1/2** | **59 (19.7%)** | **27 (19.9%)** | **32 (19.8%)** | **35 (21.9%)** | **24 (17.4%)** |
| **1** | **39 (13%)** | **23 (16.9%)** | **16 (9.9%)** | **24 (15%)** | **15 (10.9%)** |
| **<1/2 Lavash** | **15 (5%)** | **6 (4.4%)** | **9 (5.6%)** | **7 (4.4%)** | **8 (5.8%)** |
| **1 Lavash** | **144 (48%)** | **64 (47.1%)** | **80 (49.4%)** | **77 (48.1%)** | **67 (48.6%)** |
| **1 to 2 Lavash** | **10 (3.3%)** | **4 (2.9%)** | **6 (3.7%)** | **5 (3.1%)** | **5 (3.6%)** |
| **Type of meat consumed in the past year** |  |  |  |  |  |
| **Red meat** | **89 (29.7%)** | **45 (33.1%)** | **44 (27.2%)** | **46 (28.7%)** | **43 (31.2%)** |
| **Chicken** | **202 (67.3%)** | **89 (65.4%)** | **113 (69.8%)** | **112 (70%)** | **90 (65.2%)** |
| **Fish** | **7 (2.3%)** | **2 (1.5%)** | **5 (3.1%)** | **2 (1.3%)** | **5 (3.6%)** |
| **Amount of meat consumed daily (serving)** |  |  |  |  |  |
| **Equivalent to one egg** | **217 (72.3%)** | **101 (74.3%)** | **116 (73.4%)** | **121 (76.1%)** | **96 (71.1%)** |
| **Equivalent to 2 eggs** | **44 (14.7%)** | **20 (14.7%)** | **24 (15.2%)** | **24 (15.1%)** | **20 (14.8%)** |
| **More** | **33 (11%)** | **15 (11%)** | **18 (11.4%)** | **14 (8.8%)** | **19 (14.1%)** |
| **No amount** | **41 (13.7%)** | **19 (14.2%)** | **22 (13.7%)** | **22 (14%)** | **19 (13.8%)** |
| **1** | **203 (67.7%)** | **89 (66.4%)** | **114 (70.8%)** | **107 (68.2%)** | **96 (69.6%)** |
| **2** | **42 (14%)** | **23 (17.2%)** | **19 (11.8%)** | **23 (14.6%)** | **19 (13.8%)** |
| **3** | **9 (3%)** | **3 (2.2%)** | **6 (3.7%)** | **5 (3.2%)** | **4 (2.9%)** |
| **The amount of dairy consumed daily in the past year (glass)** |  |  |  |  |  |
| **< 1** | **101 (33.7%)** | **45 (33.1%)** | **56 (34.6%)** | **51 (31.9%)** | **50 (36.2%)** |
| **1** | **120 (40%)** | **49 (36%)** | **71 (43.8%)** | **62 (38.8%)** | **58 (42%)** |
| **2** | **62 (20.7%)** | **31 (22.8%)** | **31 (19.1%)** | **40 (25%)** | **22 (15.9%)** |
| **3** | **12 (4%)** | **9 (6.6%)** | **3 (1.9%)** | **5 (3.1%)** | **7 (5.1%)** |
| **More** | **3 (1%)** | **2 (1.5%)** | **1 (0.6%)** | **2 (1.3%)** | **1 (0.7%)** |
| **Type of oil consumed** |  |  |  |  |  |
| **Rapeseed oil** | **214 (71.3%)** | **96 (70.6%)** | **118 (72.8%)** | **120 (75%)** | **94 (68.1%)** |
| **Sesame Oil** | **10 (3.3%)** | **2 (1.5%)** | **8 (4.9%)** | **3 (1.9%)** | **7 (5.1%)** |
| **Olive oil** | **10 (3.3%)** | **3 (2.2%)** | **7 (4.3%)** | **5 (3.1%)** | **5 (3.6%)** |
| **Solid vegetable oil** | **54 (18%)** | **31 (22.8%)** | **23 (14.2%)** | **30 (18.8%)** | **24 (17.4%)** |
| **Animal oil** | **10 (3.3%)** | **4 (2.9%)** | **6 (3.7%)** | **2 (1.3%)** | **8 (5.8%)** |
| **Frequency of fast food consumption** |  |  |  |  |  |
| **Daily** | **3 (1%)** | **1 (0.7%)** ***** | **2 (1.2%)** | **1 (0.6%)** | **2 (1.5%)** |
| **Weekly** | **29 (9.7%)** | **5 (3.7%)** ***** | **24 (14.9%)** | **11 (7%)** | **18 (13.1%)** |
| **Monthly** | **92 (30.7%)** | **49 (36.6%)** ***** | **43 (26.7%)** | **51 (32.3%)** | **41 (29.9%)** |
| **Yearly** | **67 (22.3%)** | **30 (22.4%)** ***** | **37 (23%)** | **38 (24.1%)** | **29 (21.2%)** |
| **Never** | **104 (34.7%)** | **49 (36.6%)** ***** | **55 (34.2%)** | **57 (36.1%)** | **47 (34.3%)** |
| **Consuming all meals** |  |  |  |  |  |
| **Yes** | **240 (80%)** | **109 (80.1%)** | **131 (80.9%)** | **131 (81.9%)** | **109 (79%)** |
| **No** | **58 (19.3%)** | **27 (19.9%)** | **31 (19.1%)** | **29 (18.1%)** | **29 (21%)** |
| **Drugs history** |  |  |  |  |  |
| **Lipid-lowering** | **48 (16%)** | **32 (23.1%)** ***** | **17 (10.4%)** | **33 (20.8%)** | **16 (12.1%)** |
| **Anticoagulant** | **32 (10.7%)** | **19 (13.7%)** | **13 (8%)** | **24 (15.2%)** ***** | **8 (6.1%)** |
| **Psychiatry** | **11 (3.7%)** | **6 (4.3%)** | **5 (3%)** | **7 (4.4%)** | **4 (3.1%)** |
| **Statin** | **44 (14.7%)** | **27 (19.5%)** ***** | **17 (10.4%)** | **24 (17.4%)** | **20 (17.9%)** |
| **ACEI** | **9 (3%)** | **7 (5%)** | **2 (1.2%)** | **6 (4.3%)** | **3 (2.7%)** |
| **ARB** | **54 (18%)** | **27 (19.5%)** | **27 (16.6%)** | **27 (19.6%)** | **27 (24.1%)** |

ACEI: Angiotensin-converting-enzyme inhibitor ; ARB: Angiotensin receptor blocker. Qualitative variables is reported as frequency (percent). Quantitative variables is reported as mean ± SD.

P-value is reported based on one-way ANOVA test. *P-value is <0.05 and considered as statistically significant.

**Table 5. Biochemical indices in patients with COVID-19 in different supplement categories intake**

|  | **Total** | **Recent supplement intake** | | **Long-term supplement intake** | |
| --- | --- | --- | --- | --- | --- |
|  |  | **No** | **Yes** | **No** | **Yes** |
| **PCR test report** |  |  |  |  |  |
| **Yes** | **96 (32%)** | **46 (33.3%)** | **50 (30.87%)** | **50 (30.9%)** | **46 (33.3%)** |
| **No** | **204 (68%)** | **92 (66.7%)** | **112 (69.13%)** | **112 (69.1%)** | **92 (66.7%)** |
| **CT scan report** | **45 (15%)** | **22 (15.9%)** | **23 (14.1%)** | **26 (100%)** | **19 (100%)** |
| **AST (U/L)** | **33.61 ± 17.08** | **32.88 ± 16.86** | **34.26 ± 17.31** | **34.77 ± 17.40** | **32.13 ± 16.63** |
| **ALT (U/L)** | **33.68 ± 21.64** | **32.61 ± 19.63** | **34.61± 23.28** | **35.19 ± 22.38** | **31.73 ± 2.59** |
| **ALKPH (IU/L)** | **160.64± 109.82** | **176.96 ± 143.10** ***** | **146.64 ± 67.21** | **170.17 ± 135.89** | **148.77 ± 62.63** |
| **Ca (mg/dL)** | **8.74 ± 0.58** | **8.80 ± 0.67** | **8.69 ± 0.49** | **8.74 ±** 0.58 | **8.74 ± 0.59** |
| **P (mg/dL)** | **3.55 ± 0.66** | **3.56 ± 0.65** | **3.53 ± 0.66** | **3.52 ± 0.72** | **3.58 ± 0.57** |
| **Na (mEq/L)** | **137.49 ± 2.79** | **137.61 ± 2.77** | **137.39 ± 2.81** | **137.45 ± 2.86** | **137.54 ± 2.72** |
| **K (mmol/L)** | **4.24 ± 2.10** | **4.13 ± 0.41** | **4.34 ± 2.83** | **4.11 ± 0.37** | **4.40 ± 3.08** |
| **Mg (mg/dL)** | **1.96 ± 0.24** | **1.99 ± 0.30** | **1.93 ± 0.17** | **1.96 ± 0.18** | **1.95 ± 0.30** |
| **CPK (mcg/L)** | **166.64 ± 259.86** | **187.41 ± 343.95** | **149.56 ± 160.40** | **180.23 ± 310.84** | **149.53 ± 175.90** |
| **LDH (u/L)** | **542.85 ± 234.79** | **555.10 ± 256.03** | **532.10 ± 215.11** | **576.21 ± 239.84** ***** | **498.11 ± 221.43** |
| **FBS (mg/dL)** | **145.39 ± 80.56** | **148.34 ± 69.79** | **142.98 ± 88.57** | **149.97 ± 81.38** | **140.05 ± 79.58** |
| **HCO3 (mEq/L)** | **25.84 ± 18.67** | **25.50 ± 14.15** | **26.13 ± 21.84** | **24.17 ± 3.09** | **27.78 ± 27.16** |
| **pH** | **7.58 ± 3.31** | **7.80 ± 4.89** | **7.39 ± 0.05** | **7.76 ± 4.51** | **7.37 ± 0.26** |
| **PO2 (mmHg)** | **32.35 ± 11.48** | **33.18 ± 12.26** | **31.65 ± 10.78** | **32.33 ± 11.47** | **32.37 ± 11.55** |
| **PCO2 (mmHg)** | **42.16 ± 32.15** | **42.18 ± 34.06** | **42.14 ± 30.55** | **42.11 ± 31.70** | **42.21 ± 32.79** |
| **Troponin (ng/ml)** | **0.88 ± 0.51** | **0.88 ± 0.38** | **0.87 ± 0.60** | **0.92 ± 0.35** | **0.82 ± 0.67** |
| **WBC (10^9^/l)** | **6.98 ± 5.96** | **7.37 ± 8.14** | **6.65 ± 3.02** | **7.41 ± 7.57** | **6.49 ± 3.13** |
| **RBC (10^12^/l)** | **4.73 ± 0.63** | **4.70 ± 0.63** | **4.75 ±** **0.64** | **4.73 ± 0.60** | **4.73 ± 0.67** |
| **HCT (%)** | **40.18 ± 5.65** | **40.57 ± 5.53** | **39.86 ± 5.74** | **40.81 ± 5.23** ***** | **39.46 ± 6.03** |
| **MCV (fl)** | **85.48 ± 6.01** | **86.39 ±** 5.19 * | **84.70 ± 6.56** | **86.35 ± 5.35** ***** | **84.47 ± 6.58** |
| **MCH (pg/cell)** | **29.26 ± 2.48** | **29.46 ± 2.15** | **29.10 ± 2.72** | **29.51 ± 2.17** | **28.97 ± 2.77** |
| **Neutrophils (%)** | **73.95 ± 41.79** | **76.69 ± 60.09** | **71.60 ± 12.08** | **77.47 ± 55.71** | **69.85 ± 12.10** |
| **Lymphocyte(%)** | **23.41 ± 11.18** | **23.24 ± 11.30** | **23.57 ± 11.11** | **21.93 ± 10.90** ***** | **25.15 ± 11.29** |
| **Platelet (10^9^/L)** | **209.68 ± 80.8** | **213.74 ±** 83.43 | **206.17 ± 78.57** | **211.28 ± 82.72** | **207.80 ± 78.77** |
| **ESR (mm/hr)** | **46.31 ± 26.81** | **46.48 ±** 26.15 | **46.18 ± 27.43** | **46.92 ± 25.72** | **45.60 ± 28.13** |
| **PTT (Sec)** | **32.52 ± 8.51** | **33.09 ± 10.69** | **32.01 ±** 5.96 | **32.30 ± 7.51** | **32.77 ± 9.55** |
| **INR** | **1.04 ± 0.15** | **1.04 ± 0.16** | **1.03 ± 0.14** | **1.04 ± 0.16** | **1.03 ± 0.14** |
| **O+** | **63 (21%)** | **35 (38%)** | **28 (25.9%)** | **41 (38%)** | **22 (23.9%)** |
| **O-** | **3 (1%)** | **1 (1.1%)** | **2 (1.9%)** | **1 (0.9%)** | **2 (2.2%)** |
| **A+** | **67 (22.3%)** | **32 (34.8%)** | **35 (32.4%)** | **32 (29.6%)** | **35 (38%)** |
| **A-** | **7 (2.3%)** | **1 (1.1%)** | **6 (5.6%)** | **5 (4.6%)** | **2 (2.2%)** |
| **AB+** | **16 (5.3%)** | **5 (5.4%)** | **11 (10.2%)** | **5 (4.6%)** | **11 (12%)** |
| **AB-** | **1 (0.3%)** | **0 (0%)** | **1 (0.9%)** | **0 (0%)** | **1 (1.1%)** |
| **B+** | **39 (13%)** | **16 (17.4%)** | **23 (21.3%)** | **22 (20.4%)** | **17 (18.5%)** |
| **B-** | **4 (1.3%)** | **2 (2.2%)** | **2 (1.9%)** | **2 (1.9%)** | **2 (2.2%)** |

PCR: Polymerase chain reaction; CT: computed tomography; BUN: blood urea nitrogen; AST: Aspartate transaminase; ALT: Alanine transaminase; ALKPH: Alkaline Phosphatase; CPK: Creatine Phosphokinase LDH: Lactate Dehydrogenase; FBS: Fasting blood sugar; PO2: partial pressure of oxygen; PCO2: partial pressure of carbon dioxide; CRP: C-Reactive Protein; TG: Triglyceride; WBC: White blood cell; RBC: red blood cell; Hb: Hemoglobin; HCT: hematocrit; MCV: mean corpuscular volumeMCH; ESR: Erythrocyte sedimentation rate; PTT: partial thromboplastin time; INR: international normalized ratio. Qualitative variables is reported as frequency (percent). Quantitative variables is reported as mean ± SD. P-value is reported based on one-way ANOVA test.

*P-value is <0.05 and considered as statistically significant.
